# The Impact of Wearable Technologies in Health Research: Scoping Review

**DOI:** 10.2196/34384

**Published:** 2022-01-25

**Authors:** Sophie Huhn, Miriam Axt, Hanns-Christian Gunga, Martina Anna Maggioni, Stephen Munga, David Obor, Ali Sié, Valentin Boudo, Aditi Bunker, Rainer Sauerborn, Till Bärnighausen, Sandra Barteit

**Affiliations:** 1 Heidelberg Institute of Global Health Heidelberg University Hospital Heidelberg University Heidelberg Germany; 2 Charité - Universitätsmedizin Berlin Institute of Physiology Center for Space Medicine and Extreme Environment Berlin Germany; 3 Department of Biomedical Sciences for Health Università degli Studi di Milano Milano Italy; 4 Kenya Medical Research Institute Kisumu Kenya; 5 Centre de Recherche en Santé Nouna Nouna Burkina Faso; 6 Harvard Center for Population and Development Studies Cambridge, MA United States; 7 Africa Health Research Institute KwaZulu-Natal South Africa

**Keywords:** wearable, consumer-grade wearables, commercially available wearables, public health, global health, population health, fitness trackers, big data, low-resource setting, tracker, review, mHealth, research, mobile phone

## Abstract

**Background:**

Wearable devices hold great promise, particularly for data generation for cutting-edge health research, and their demand has risen substantially in recent years. However, there is a shortage of aggregated insights into how wearables have been used in health research.

**Objective:**

In this review, we aim to broadly overview and categorize the current research conducted with affordable wearable devices for health research.

**Methods:**

We performed a scoping review to understand the use of affordable, consumer-grade wearables for health research from a population health perspective using the PRISMA-ScR (Preferred Reporting Items for Systematic Reviews and Meta-Analyses extension for Scoping Reviews) framework. A total of 7499 articles were found in 4 medical databases (PubMed, Ovid, Web of Science, and CINAHL). Studies were eligible if they used noninvasive wearables: worn on the wrist, arm, hip, and chest; measured vital signs; and analyzed the collected data quantitatively. We excluded studies that did not use wearables for outcome assessment and prototype studies, devices that cost >€500 (US $570), or obtrusive smart clothing.

**Results:**

We included 179 studies using 189 wearable devices covering 10,835,733 participants. Most studies were observational (128/179, 71.5%), conducted in 2020 (56/179, 31.3%) and in North America (94/179, 52.5%), and 93% (10,104,217/10,835,733) of the participants were part of global health studies. The most popular wearables were fitness trackers (86/189, 45.5%) and accelerometer wearables, which primarily measure movement (49/189, 25.9%). Typical measurements included steps (95/179, 53.1%), heart rate (HR; 55/179, 30.7%), and sleep duration (51/179, 28.5%). Other devices measured blood pressure (3/179, 1.7%), skin temperature (3/179, 1.7%), oximetry (3/179, 1.7%), or respiratory rate (2/179, 1.1%). The wearables were mostly worn on the wrist (138/189, 73%) and cost <€200 (US $228; 120/189, 63.5%). The aims and approaches of all 179 studies revealed six prominent uses for wearables, comprising correlations—wearable and other physiological data (40/179, 22.3%), method evaluations (with subgroups; 40/179, 22.3%), population-based research (31/179, 17.3%), experimental outcome assessment (30/179, 16.8%), prognostic forecasting (28/179, 15.6%), and explorative analysis of big data sets (10/179, 5.6%). The most frequent strengths of affordable wearables were validation, accuracy, and clinical certification (104/179, 58.1%).

**Conclusions:**

Wearables showed an increasingly diverse field of application such as COVID-19 prediction, fertility tracking, heat-related illness, drug effects, and psychological interventions; they also included underrepresented populations, such as individuals with rare diseases. There is a lack of research on wearable devices in low-resource contexts. Fueled by the COVID-19 pandemic, we see a shift toward more large-sized, web-based studies where wearables increased insights into the developing pandemic, including forecasting models and the effects of the pandemic. Some studies have indicated that big data extracted from wearables may potentially transform the understanding of population health dynamics and the ability to forecast health trends.

## Introduction

### Background

Wearable devices hold great promise, particularly for data generation for cutting-edge health research, and their demand has risen considerably in the last few years [1-3].

Noninvasive, consumer-grade wearables (hereafter *wearables*) may provide manifold advantages for health research; they are generally unobtrusive, less expensive than gold standard research devices [4], comfortable to wear [5], and affordable for consumers [6]. In recent years, the quality and accuracy of wearables have improved [7,8], resulting in more clinically approved certifications [9]. Wearables can measure long-term data in the naturalistic environment of study participants, allowing for ecologic momentary assessments [10,11]. Therefore, wearables are valuable developments, particularly for generating data for health research in large study populations, that is, global health or epidemiological studies, or in low-income contexts [6,9,12].

One example of a large study is the so-called *Datenspende* study by the Robert Koch Institute, the German research institute for disease control and prevention, which aims to tackle the COVID-19 (corona virus disease) pandemic with anonymous data donations acquired through wearables [13]. On the basis of the study by Radin et al [14], researchers used wearable data to calculate the regional probability of COVID-19 outbreaks incorporating data on pulse, physical activity (PA), and sleep, as well as weather data. Using a large sample size exceeding half a million participants, they forecasted the number of COVID-19 infections for the preceding 4 days. The Apple Heart Study [15] is another example that was a breakthrough for showing that wearable devices may detect atrial fibrillation (AF) and foster a discussion of potentials and limitations with regard to health care providers, researchers, and members of the media and economy [16,17].

Apart from these 2 examples, wearables are applied in diverse fields of health, including acoustic, gastrointestinal sensors for ileus prediction [18]; UV sun exposure [19]; heat-related illness measurements [20]**;** electrolyte monitoring, for example, for cystic fibrosis or training management [21,22]; early warning of AF with a wearable ring [23]; generation of electrocardiograms (ECGs) [15]; measurement of cardiopulmonary resuscitation quality [24]; measurement of continuous noninvasive blood glucose [25], as well as smart inhalers and activity trackers for asthma monitoring [26].

Numerous reviews and studies have investigated validation and accuracy, particularly for specific affordable wearables, comparing these to the gold standard measurements [21] or comparing evidence in a meta-analysis [8]. Many studies have focused on novel technologies, presenting prototypes, or investigating the feasibility and acceptance of a wearable device in a specific setting [3,27]. Similarly, reviews on the application and potential of wearables have focused on (1) specific wearable devices or specific wearable measurements, for example, only smartwatches [4] or only sleep measurements [28] or (2) applications of specific medical fields and interventions, for example, only for diagnosis and treatment in cardiological conditions [29] or wearables as an intervention to promote PA in patients with oncologic conditions [30]. Among these publications, we identified a lack of aggregated insight for wearable use in health research and its respective strengths and shortcomings.

### Objectives

With this scoping review, we aim to overview and categorize the current research conducted on wearable devices.

## Methods

### Overview

We conducted a scoping review to explore the applications of affordable wearables worn on wrists, arms, chests, or waists, which constitute the characteristic locations [31]. We focused on the following aspects: (1) demographics; (2) wearable devices and measured vital signs; (3) wearable data and its analysis; (4) reported shortcomings and strengths of wearables; and (5) study aims, results, and types of wearable use. We present our findings in accordance with the PRISMA (Preferred Reporting Items for Systematic Reviews and Meta-Analyses) reporting standard and PRISMA-ScR (Preferred Reporting Items for Systematic Reviews and Meta-Analyses extension for Scoping Reviews; Multimedia Appendix 1) [32] and the methodological framework of Arksey and O’Malley [33] and Peters et al [34]. A scoping review seemed most appropriate given the broad nature of this subject and the range of potential implementations in the setting of health research.

### Eligibility Criteria

We sought to define and characterize the state of affordable wearables for health research. Eligible publications were peer reviewed, published in English, and published after 2013 (after wearables became widely commercially available [1-3]) and had a full-text version available (in instances no full text was available, authors were contacted 3 times with a waiting period of 7 days between each contact before exclusion).

Our review scopes the current information available on affordable, noninvasive wearables, which are (1) worn on the wrist, arm, and chest; (2) measure vital signs; and (3) analyze the generated wearable data for outcome assessment. Validation and qualitative studies were excluded. We focused only on devices that cost <€500 (US $570) per device (1) to allow the affordability of larger studies, for example, where wearable devices need to be provided to study participants via the study and (2) to ensure that wearables are available commercially and (3) intended for consumers. As the definition of vital signs is not distinct [35], we included the following vital signs [9,36,37]: HR, HR variability, ECG measurements or heart rhythm analysis (detection of arrhythmias), blood pressure, blood oxygen, respiratory rate, body temperature, sleep, electrodermal activity, electromyogram measurements, and PA (Textbox 1).

**Inclusion and exclusion criteria.**

**Inclusion criteria**

- Publications
  Full text available
  English language
  Peer-reviewed articles
  Published between 2013 and 2020
- Wearable device
  Commercially available wearable, price <€500 (US $570) per device (Only hardware prices were considered. Software, subscriptions, or similar, which might be necessary for device use, were not included. All prices were captured in the timeframe of this study and therefore are only considered as approximations)
  Wearables worn on the arm, wrist, chest, and waist
- Outcomes
  Measuring and analyzing one or more vital sign
  Range of vital signs as defined in this review, including heart rate, heart rate variability, electrocardiogram measurements or heart rhythm analysis (detection and classification of atrial fibrillation, extrasystoles, and other arrhythmic events), blood pressure, blood oxygen, respiratory rate, body temperature, sleep (time, deepness, etc), electrodermal activity, electromyogram measurements, physical activity (steps, distance covered, intensity, energy expenditure, etc; physical activity included as basic measurements of wearables or very similar or related parameters) [9,36,37].

**Exclusion criteria**

- Publications
  Studies not analyzing wearable-generated data for (health) outcome assessment, including studies focusing on (1) accuracy, validation, improvement (algorithms and software); (2) patents; (3) smart clothing; (4) obtrusive wearables (the device comprises obstructive parts or wires, etc); (5) behavior change intervention studies (ie, where the wearable is provided as promotion for more physical activity only and not for health outcome assessment); (6) qualitative studies; or (7) studies with research objectives and outcomes not related to health or a medical condition
- Wearable device
  Wearable not commercially available (eg, prototype and discontinued)
  Invasive, obtrusive device (comprising obstructive parts or wires, etc)
  Prosthesis, smart clothing (sensors in clothing)
- Outcomes
  Not measuring vital sign, that is, gait, posture, and motion recognition analysis (eg, gesture recognition for sign language)
  Studies with research objectives and outcomes not related to health or a medical condition

### Information Sources and Search

We used PubMed, Ovid, Web of Science, and CINAHL to search peer-reviewed literature using a search string based on the following three concepts: synonyms and medical subject headings terms, including (1) wearables (synonyms, top 15 vendors with most market shares [38-40], or frequently used in research [2,7]), (2) physical wear location of wearables (torso, arm, and wrist), and (3) measurement of vital signs (for full search string see Multimedia Appendix 2 [41]). We manually searched the reference lists for relevant articles.

We imported the identified articles into the literature reference management system Zotero [42] and then into the systematic review management platform Covidence [41]. Literature was screened by 2 independent reviewers. Any disagreements were resolved by discussion between the 2 reviewers (SH and MA) and a third researcher (SB).

### Quality Assessment

To assess the quality of the included studies and their various study designs (credibility), we considered the Medical Education Research Study Quality Instrument [43] score as adequate (Multimedia Appendix 3 [14,15,20,44-219]).

### Data Synthesis

We conducted data synthesis in accordance with Arksey and O’Malley [33], comprising the analytic framework, analysis of the extent and nature of studies, and thematic analysis. We categorized the findings by title, author, year, country of study, objectives of study, study population, sample size, methods, intervention type, outcomes, and key findings related to the scoping review question [34]. We extracted mutually exclusive groups, including wearable manufacturers, built-in sensors, scope of measurements (vital signs), shortcomings and strengths of wearables mentioned by the authors, the used methods for data analysis, and medical fields.

## Results

### Overview

**Figure 1:**
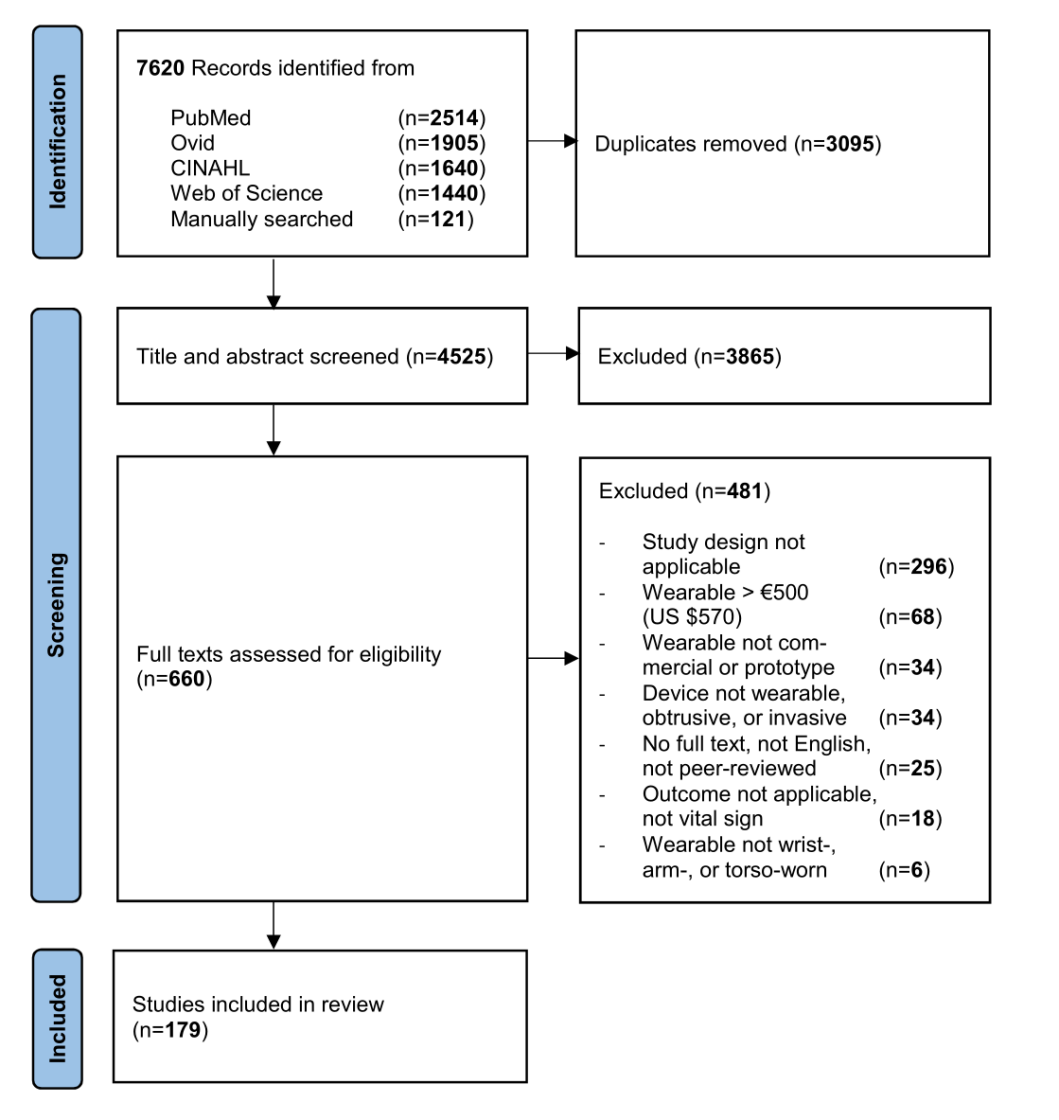
PRISMA (Preferred Reporting Items for Systematic Reviews and Meta-Analyses) flow diagram [220].

Our initial search yielded 7499 hits (PubMed: 2514; Ovid: 1905; Web of Science: 1440; CINAHL: 1640) and we identified 121 publications by manual search. Of 7620 total publications, we screened 4525 (59.38%) nonduplicates for title and abstract, leading to the assessment of 660 full-texts. After full-text screening of the 660 articles, we included 179 (27.1%) studies in our review [14,15,20,44-219] (Figure 1).

### Study Characteristics

#### Demographics

**Figure 2:**
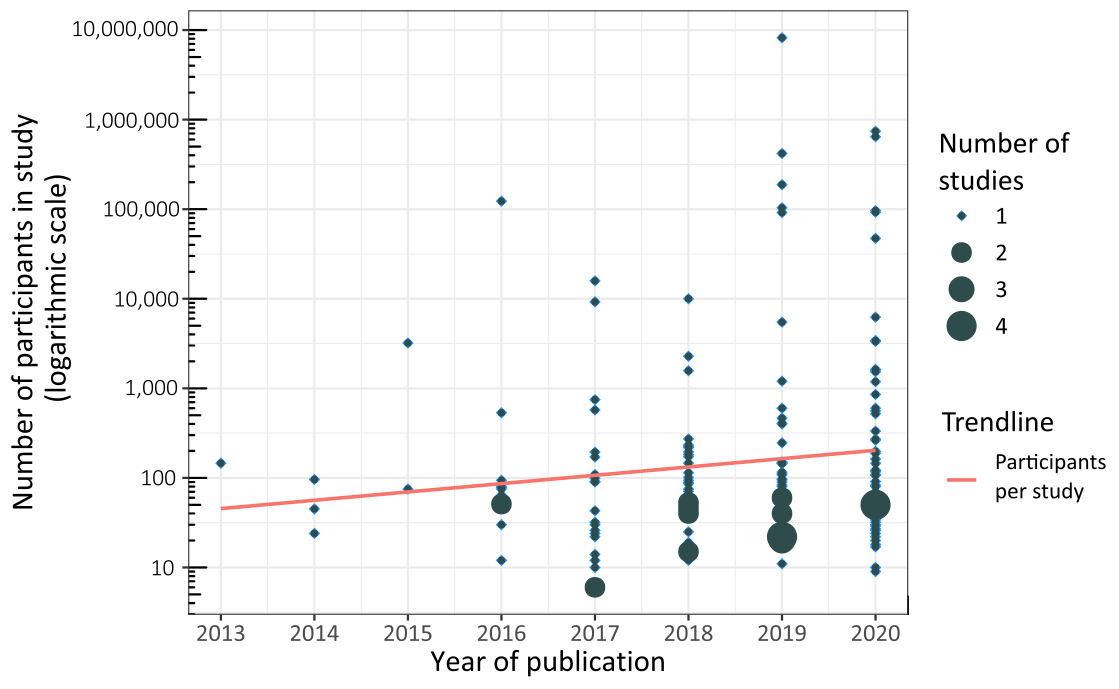
Number of studies and study participants (logarithmic scale) per year of study publication. The sizes of the circles visualize the overlapping and number of studies within the year.

**Figure 3:**
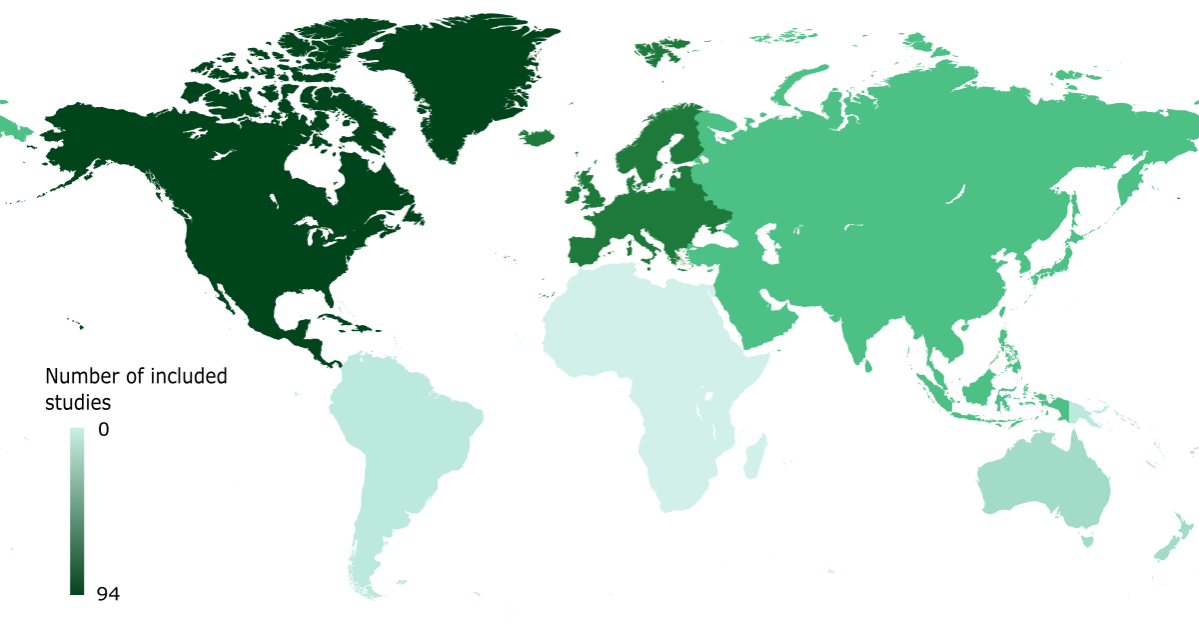
Included studies per continent. The colors of the continents visualize the number of included studies published on the respective continent (created with Mapchart [221]).

Between 2013 and 2020, we observed an increase in the number of studies and study participants (Figure 2 and Table 1). The year 2019 featured the largest sample size, and studies were predominantly conducted in North America (Figure 3 [221]). The largest study we identified was conducted in 2019 in North America and included over 8 million participants (75.71%) [153]; the second largest was a European study comprising 742,000 participants (6.85%) [162]. Without the aforementioned, largest study, Europe and Asia would lead in participant numbers and we would see a continuous increase in participant numbers from 2013 to 2020.

**Table 1:** Characteristics of studies.

Study characteristics		Studies (N=179), n (%)	Participants (N=10,835,733), n, (%)
**Year of publication**			
	2013	1 (0.56)	146 (<0.01)
	2014	3 (1.68)	165 (<0.01)
	2015	2 (1.12)	3284 (0.03)
	2016	14 (7.82)	124,060 (1.14)
	2017	21 (11.73)	27,377 (0.25)
	2018	34 (18.99)	16,700 (0.15)
	2019	48 (26.82)	9,016,909 (83.21)
	2020	56 (31.28)	1,647,092 (15.2)
**Continents**			
	North America	94 (52.51)	8,916,888 (82.29)
	Europe	50 (27.93)	991,357 (9.15)
	Asia	24 (13.41)	925,768 (8.54)
	Australia	8 (4.47)	1198 (0.01)
	South America	3 (1.68)	522 (<0.01)
**Study objectives**			
	Correlations and influencing factors of study population and outcome data^a^	70 (39.11)	394,296 (3.64)
	Population and patient characterization^b^	54 (30.17)	8,315,559 (76.74)
	Evaluation of method or intervention	47 (26.26)	2,124,328 (19.6)
	Prognostic evaluation^c^	8 (4.5)	1550 (0.01)
**Study design**			
	Cross-sectional study	66 (36.87)	9,780,808 (90.26)
	Cohort study	62 (34.64)	628,641 (5.8)
	Nonrandomized experimental study	14 (7.82)	724 (0.01)
	Randomized controlled trial	11 (6.15)	2332 (0.02)
	Method evaluation	8 (4.47)	314,247 (2.9)
	Other	7 (3.91)	108,462 (1)
	Case control study	7 (3.91)	348 (<0.01)
	Mixed methods, feasibility study	4 (2.23)	171 (<0.01)
**Medical field of study**			
	Multidisciplinary and general medicine	43 (24.02)	107,148 (0.99)
	Neurology and psychiatry	29 (16.2)	2630 (0.02)
	Cardiology, fitness, and sports medicine	28 (15.64)	557,120 (5.14)
	Global health, epidemiology, and prevention	19 (10.61)	10,104,217 (93.25)
	Gynecology and pediatrics	18 (10.06)	5575 (0.05)
	Orthopedics and surgery	16 (8.94)	2749 (0.03)
	Pulmonology	13 (7.26)	1326 (0.01)
	Other	13 (7.26)	54,968 (0.51)

^a^Studies aimed to find associations, correlations, or influencing factors within their study population, study outcomes, and generated data.

^b^Studies aimed to observe and characterize the study population and patients.

^c^Studies aimed to evaluate patient-reported outcomes, health care practices, diagnostics, screenings, and others.

#### Study Types and Fields

Most studies (128/179, 71.5%) used observational study designs such as cross-sectional (66/179, 36.9%) and cohort studies (62/179, 34.6%), comprising 9,780,808 (90.26%) participants and 628,641 (5.8%) participants, out of 10,835,733 participants, respectively. Most frequently, studies (70/179, 39.1%) aimed to find associations, correlations, or influencing factors within their study population, study outcomes, and generated data. Slightly less than one-third of the studies (54/179, 30.2%) aimed to characterize and observe their study population.

**Figure 4:**
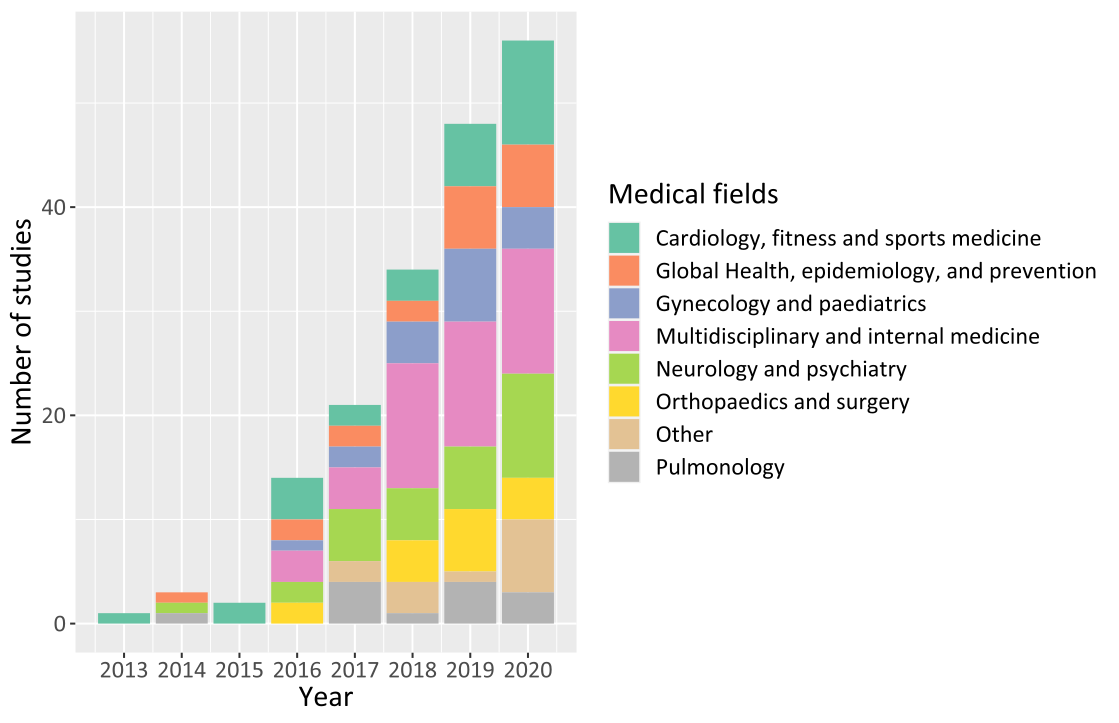
Studies per medical field.

Most studies were conducted in the fields of multidisciplinary and general medicine (43/179, 24%); cardiology, fitness, and sports medicine (29/179, 16.2%); and neurology, psychology, and psychiatry (28/179, 15.6%; Figure 4). The fields of global health, prevention, and epidemiology featured the largest sample size with, with 10,104,217 (93.25%) out of 10,835,733 participants.

### Wearable Characteristics

A total of 189 wearable devices were extracted. The company with the most wearable devices in the included studies was Fitbit (97/189, 51.3%), covering 8,361,035 (74.35%) out of 11,224,872 participants. Fitbit is followed by ActiGraph (research-grade wearable devices unavailable for consumers or not consumer grade per se; 19/189, 10.1%), Polar Electro (9/189, 4.8%), and Withings (8/189, 4.2%). In number of study participants, Huawei and Withings comprised 832,036 (7.4%) participants and 794,174 (7.06%) participants out of 11,224,872 participants, respectively (Table 2).

**Table 2:** Characteristics of wearable devices.

Wearable characteristics		Studies (N=189), n (%)	Participants (N=11,244,872), n (%)
**Wearable companies used in studies**			
	Fitbit	97 (51.32)	8,361,035 (74.35)
	ActiGraph^a^	19 (10.05)	2571 (0.02)
	Polar electro	9 (4.76)	6970 (0.06)
	Withings	8 (4.23)	794,174 (7.06)
	iRhythm	6 (3.17)	128,641 (1.14)
	Xiaomi	5 (2.65)	176 (<0.01)
	Axivity^a^	4 (2.12)	291,871 (2.6)
	Garmin	4 (2.12)	308 (<0.01)
	Apple	4 (2.12)	420,826 (3.74)
	Activinsights^a^	3 (1.59)	1971 (0.02)
	Samsung	2 (1.06)	120 (<0.01)
	Ava AG	2 (1.06)	285 (<0.01)
	Huawei	2 (1.06)	832,036 (7.40)
	Whoop	2 (1.06)	305 (<0.01)
	Omron	2 (1.06)	159 (<0.01)
	Other companies (wearable only included in 1 study)	20 (10.58)	423,424 (3.77)
**Number of wearable device models per study (n=179)**			
	1	156 (87.15)	486,684 (4.49)
	2	11 (6.15)	420,007 (3.88)
	3	3 (1.68)	838,266 (7.74)
	>3 or not applicable^b^	9 (5.03)	9,090,776 (83.9)
**Wearable device types**			
	Fitness tracker	86 (45.5)	22,823 (0.2)
	Accelerometer (worn on wrist, torso, and hip)	49 (25.93)	299,251 (2.66)
	Electrocardiogram chest patch or strap	21 (11.11)	530,332 (4.72)
	Smartwatch	12 (6.35)	1,259,605 (11.2)
	Diverse wearable devices—secondary data via wearable data platform	11 (5.82)	9,122,758 (81.13)
	Distinct vital sign trackers (eg, oximetry ring, temperature wristband tracker, and blood pressure armband)^c^	10 (5.29)	10,103 (0.09)
**Physical location of wearable**			
	Wrist	138 (73.02)	10,702,843 (95.18)
	Hip	25 (13.23)	2257 (0.02)
	Chest	21 (11.11)	550,332 (4.89)
	Arm	3 (1.59)	9392 (0.08)
	Finger	2 (1.06)	48 (<0.01)
**In studies used in-built sensor in wearables^d^** **(n=179)**			
	Accelerometer	146 (81.56)	1,157,069 (10.68)
	Photoplethysmography	59 (32.96)	9,622,147 (88.8)
	Electrodes (ie, electrocardiogram)	21 (11.73)	550,500 (5.08)
	Gyroscope	6 (3.35)	1585 (0.01)
	Thermometer	4 (2.23)	842 (0.01)
	Blood pressure sensor	3 (1.68)	9397 (0.09)
**Wearable costs (€; US $)**			
	<200 (228)	120 (63.49)	340,460 (3.03)
	200-350 (228-399)	41 (21.69)	18,256 (0.16)
	>350 (399)	13 (6.88)	551,128 (4.9)
	Not applicable^e^	15 (7.94)	10,355,028 (92.09)
**Analysis—statistical tests^f^** **in studies (n=179)**			
	Regression	62 (34.64)	1,021,032 (9.42)
	*t* test	41 (22.91)	8,309,202 (76.68)
	Correlation (Pearson, Spearman, etc)	40 (22.35)	11,044 (0.1)
	Wilcoxon *U*, Mann–Whitney *U*, and other nonparametric tests	23 (12.85)	7180 (0.07)
	Chi-square and Fisher–Yates tests	15 (8.38)	433,785 (4)
	Mixed methods model and other statistical models	14 (7.82)	57,938 (0.53)
	Artificial Intelligence (data mining, cluster, machine learning, etc)	11 (6.15)	835,967 (7.71)
	Analysis of variance	11 (6.15)	810 (0.01)
	Descriptive	8 (4.47)	423,093 (3.9)
	Prognostic analysis (Kaplan–Meier, permutation test, etc)	3 (1.67)	420,928 (3.88)

^a^Research-grade wearable devices unavailable for consumers or not consumer grade per se.

^b^Studies collected data with multiple wearable devices (that belonged to the study participants) or studies that used secondary data provided by web-based wearable platforms, mobile applications, or wearable companies.

^c^Distinct vital sign trackers are specialized on a specific vital sign, for example, oximetry ring, temperature wristband tracker, and blood pressure armband. They differ in measured vital signs and worn locations compared with other wearable device types.

^d^Utilized in-built sensors in wearables sums up to more than the total of wearables, as sometimes more than one built-in sensor was used.

^e^Providing wearable hardware pricing was not transparent, as some studies used data provided by diverse participant-owned wearables or wearable hardware costs were part of a subscription or a membership fee, that is, Whoop strap of Whoop.

^f^Analysis—statistical tests sums up to more than the total number of included studies, as some studies applied more than one type of analysis or statistical test.

Most studies (156/179, 87.2%) used 1 wearable model. However, most of the study participants (9,090,776/10,835,733, 83.9%) were part of large-scale population-based studies in which data were mostly collected with multiple wearable devices that belonged to the study participants.

Some large-scale population-based studies (11/179, 6.1%) relied on secondary data collected with mobile apps [87] or web-based wearable platforms [153] or provided through a wearable company [189]. Thus, the device type could not be specified (assigned to category *diverse wearable devices—secondary data via wearable data platform*). A total of 15 (63%) out of 24 studies that used secondary data were conducted in 2020, and 5 (21%) studies in 2019.

Fitness trackers (86/189, 45.5%) and accelerometers (measuring body movement acceleration [37]) worn on the wrist, torso, and hip (49/189, 25.9%) were the most frequent. Other wearable device types included ECG chest straps and patches (21/189, 11.1%), smartwatches (12/189, 6.3%), and distinct vital sign trackers (10/189, 5.3%) such as oximetry rings or blood pressure armbands (Table 2).

**Figure 5:**
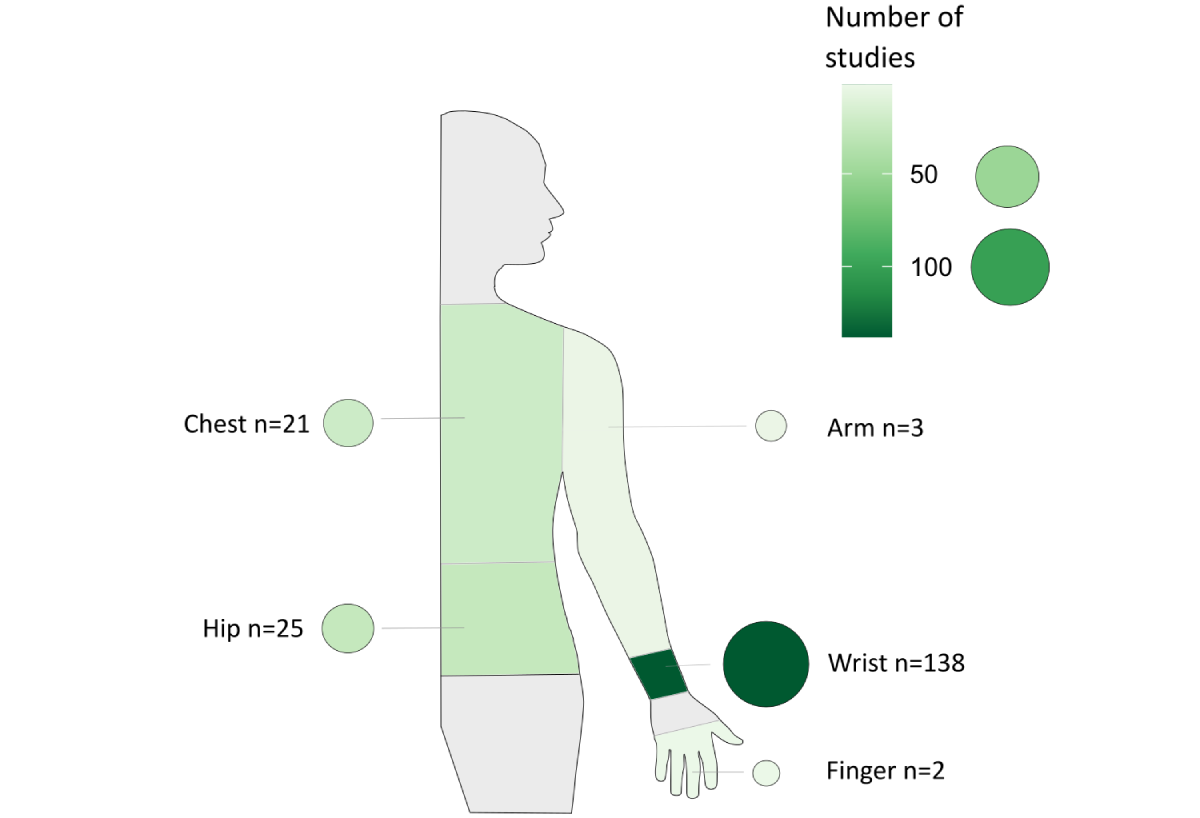
Wear locations of wearables and their frequencies. The color and size of the circles assigned to the body location visualize the frequency of wearables worn on the respective location.

Most wearables were worn on the wrist (138/189, 73%), followed by the hip (25/189, 13.2%) and chest (21/189, 11.1%). Only a few wearables were worn on the arm (3/189, 1.6%) and finger (2/189, 1.1%; Figure 5).

Most of the studies used wearable built-in sensors of (1) accelerometers (146/179, 81.6%) that measure acceleration on a 3- or 1-axis [37] and (2) photoplethysmography (59/179, 33%) defined as an “optical technique that [...] detects blood volume changes in the microvascular bed of tissue” [222]. Other built-in sensors were electrodes for ECG measurements (21/179, 11.7%); gyroscopes (6/179, 3.4%), which determine how different portions of the body rotate [37]; thermometers (4/179, 2.2%) measuring skin temperature; and blood pressure sensors (3/179, 1.7%).

Most studies investigated steps (95/179, 53.1%), HR (55/179, 30.7%), and sleep time (51/179, 28.5%). We classified measured vital signs into three categories, whereby PA measures were most frequent (228/179, 127.4%; Multimedia Appendix 4 [14,15,20,44-219]):

1. PA measures included steps, intensity (eg, time spent in moderate to vigorous PA), energy expenditure (eg, kilocalories and metabolic equivalent), axial or raw movement data, distance (covered), and others (such as stairs taken, elevation, and sedentary time).
2. Cardiac measures included HR, HR variability, and ECG (or other direct heart rhythm analyses, such as AF detection).
3. Other measures that included blood or pulse pressure, body temperature, blood oxygen, and respiratory rate.

Most studies (120/189, 63.5%) used wearables that cost <€200 (US $228). In some studies (15/189, 7.9%), wearable prices were not transparent, as data were provided through a variety of participant-owned wearables [87] or the wearable hardware was part of a subscription or a membership fee, that is, Whoop strap of Whoop [178].

Regression analysis (62/179, 34.6%) and *t* tests (42/179, 22.9%) were the most commonly used statistical methods to analyze wearable data. Other methods comprised nonparametric tests, such as correlations, Wilcoxon U test, Kaplan-Meier survival analysis, and chi-square tests. Variance analysis (analysis of variance) and significance tests such as permutations were also used. Further data analyses were conducted in a data-driven manner [223] with artificial intelligence, such as k-means [176] or unsupervised cluster analysis [172], recursive feature elimination technique [170], rotation random forest classifier [130], and supervised machine learning algorithms using logistic regression, decision tree, and random forest [215].

### Categorization of Wearable Application in the Studies

**Figure 6:**
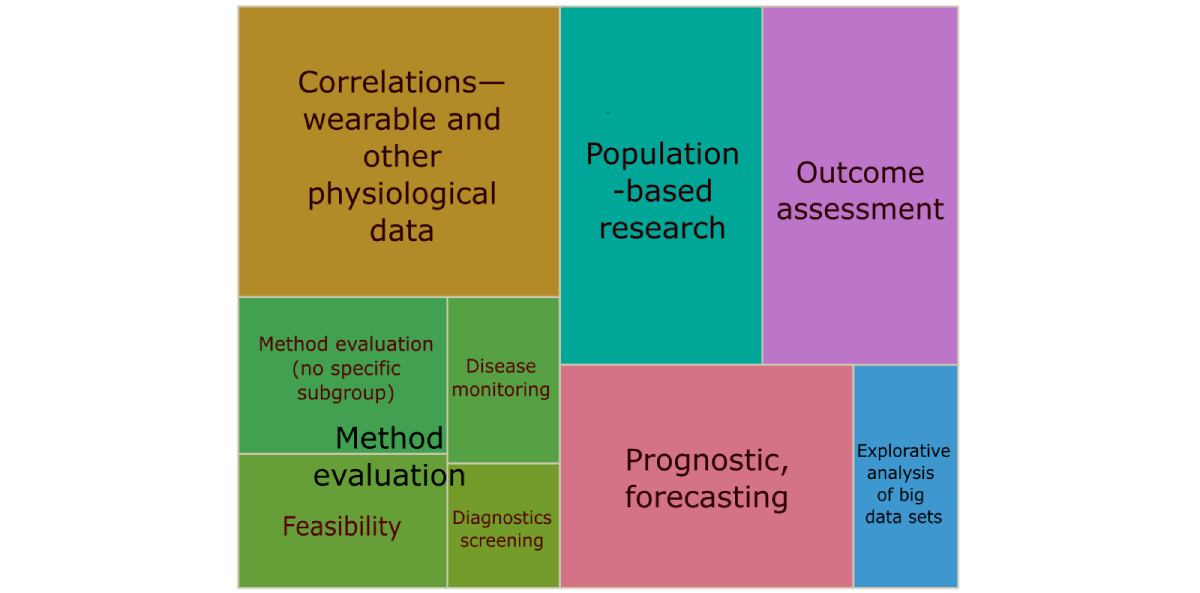
Categorization of wearable applications, showing proportions of the 6 categories (with 4 subcategories). The size of depicted categories (in different colors) corresponds to the number of studies.

We categorized the included studies based on their study objective, the role of the wearable and the collected wearable data within the study in the following 6 categories (overlaps are possible as separation is artificial). In the following, categories are presented in order of their frequency (see Figure 6 and Multimedia Appendix 5 [14,15,20,44-219] for article references and examples).

#### Correlations—Wearable and Other Physiological Data

Studies (40/179, 22.3%) have examined the correlation of a wearable derived measure with clinical- and patient-reported and other health-related outcomes to find new associations and correlations. The data generated by the wearable device were correlated with data from mostly physiological or patient-reported outcomes.

#### Population-Based Research

In 17.3% (31/179) of studies, wearables produced insights into a specific population through monitoring (observational and cross-sectional) of vital signs, such as steps and HR. Often, these were cross-sectional studies (17/31, 55%) where the wearable measurement was the sole outcome. The resulting data provide novel insights and characteristics of populations.

#### Outcome Assessment

In these studies (30/179, 17.3%), wearables generated the outcome measurement and monitored the dependent variable in an (quasi-) experimental setting or intervention, in mostly randomized controlled trials and quasi-experimental designs.

#### Prognosis, Forecasting, and Risk Stratification

In further studies (28/179, 15.6%), data generated with wearables were integrated into risk calculations (risk for a certain event or outcome), prognostic models, or cut-points. Wearable data constituted inputs for models to estimate risks.

#### Explorative Analysis of Big Data Sets

These studies (10/179, 5.6%) exploratively analyzed big data [223], generated by wearables and accessible via applications, commercial platforms, eCohorts, or companies themselves, to find trends and generate new hypotheses.

#### Method Evaluation

Studies (40/179, 22.3%) have evaluated and compared methods and tools (such as screenings for diseases, general practices, questionnaires, or other patient-reported outcomes) with the help of wearables. The wearable device might be the gold standard device or probed itself.

#### Feasibility

In these studies (12/179, 6.7%), the feasibility of using wearables for screening diseases and to improve on existing methods and practices is focused, mostly accompanied by a qualitative component.

#### Diagnostics and Screening

Studies (6/179, 3.4%) in this category evaluated details of diagnostics and disease screening outcomes, (cost-) effectiveness, utility, and screening length or were compared with standard measurement methods.

#### Disease Monitoring

Here (8/179, 4.5%), wearables supported the monitoring of an already diagnosed condition or a patient at risk (of deterioration).

#### Others

Studies (14/179, 7.8%) evaluated methods, with no other particular subgroup being appropriate.

### Strengths and Shortcomings of Wearables

Overall, the studies mentioned more strengths than shortcomings. A few studies (16/179, 8.9%) mentioned no strengths of wearables, whereas 55.3% (99/179) of the studies mentioned no shortcomings.

**Figure 7:**
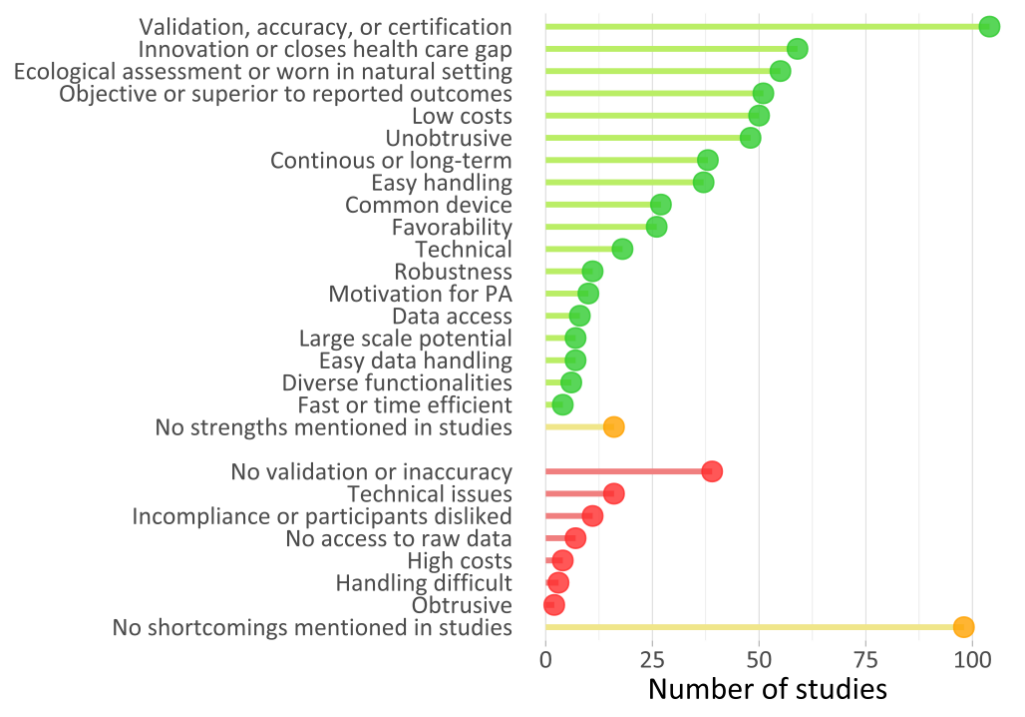
Chart of reported strengths and weaknesses of wearables as mentioned by authors. PA: physical activity.

Most often, authors (104/179, 58.1%) emphasized the accuracy and reliability, positive results of peer-reviewed validation studies (own and of others), or clinically approved certifications (eg, the Food and Drug Administration [FDA] clearance in the United States or Communauté Européenne [CE] mark of the European Union; Figure 7).

Often, studies (59/179, 33%) identified the wearable as innovative, that is, as a cutting-edge tool and method [103] with a wearable device potentially closing a gap in or improving health care and research. For example, 1 study described how wireless wearables and data synching could improve the quality of care [69], “The data can be sent from the wearable to the physician’s office, avoiding the need for office visits, ultimately making possible preventive medicine and improving quality of care.” Low et al [129] concluded that “Fitbit devices may provide opportunities to improve postoperative clinical care with minimal burden to patients or clinical providers.” Tomitani et al [199] reflected how wrist-worn blood pressure wearables could “significantly improve blood pressure control.” As per Shilaih et al [184], wrist-worn wearables might ameliorate fertility awareness research and care.

Several studies (55/179, 30.7%) acknowledged the ability of wearables to measure in the naturalistic environment of the participants, called *ecological momentary assessment* [10,11,224].

Multiple studies (51/179, 28.5%) described wearables as objective and superior to self-reported outcomes as they were more accurate, reliable, and easier to generate. Often, the authors valued the relatively low costs of wearables (50/179, 27.9%). Others appreciated wearables as being unobtrusive or noninvasive (48/179, 26.8%) and enabling continuous, long-term measurements (38/179, 21.2%). Furthermore, the handling (37/179, 20.7%) of hardware and software was often found to be user-friendly, as well as the prevalence of wearables in the population (27/179, 15.1%), decreasing stigma and easing participant recruitment. Some studies (26/179, 14.5%) reported that participants accepted and liked the wearables, resulting in high participant compliance (wearing and using the wearable). Some authors (18/179, 10.1%) perceived technical wearable characteristics as positive, for example, good sampling rate of measurements, long battery life, large memory space, raw data availability, data security, compatibility with other devices such as smartphones, and availability of application programing interfaces (APIs).

Few studies (11/179, 6.1%) described wearables as robust and not easy to break. Authors (10/179, 5.6%) valued the wearable-induced behavior change as a cobenefit, that is, motivating study participants to more PA and increasing health awareness.

A few studies (8/179, 4.5%) mentioned data accessibility via APIs, apps, and web-based platforms and a few other studies (7/179, 3.9%) potential of large-scale wearable studies, or the ease of data handling. A few (6/179, 3.4%) studies underlined the variety of functionalities and vital sign measurements as positive aspects, and 2.2% (4/179) of studies perceived wearables as fast or time-efficient in data generation.

Most shortcomings (39/179, 21.8%) were related to the inaccuracy of the wearables or the absence of validation or clinically approved certification. Studies (16/179, 8.9%) also mentioned technical issues, such as a low sampling rate of measurements, no wear time recognition, or missing data. Other technical issues comprised, for example, synchronization, charging and device setup [91] or data cleaning [137]. Rare experienced shortcomings were participants’ noncompliance or dislike toward the wearable (11/179, 6.1%), no access to raw data or company’s algorithms (4/179, 2.2%), difficulties in handling the wearable (3/179, 1.7%), and wearables perceived as obtrusive in daily life (2/179, 1.1%).

## Discussion

### Study Characteristics

Overall, we have identified a positive trend in wearable studies, underlining the growing interest in wearables in health research, in line with other reviews [3,224-226]. Our results show a strong interest of researchers and study participants in this technology, but we also identified cautionary behavior toward using wearables. The vast majority of studies were undertaken in North America, about twice as many as in Europe, which is consistent with the previous literature [225]. One study in North America, conducted in 2019 with over 8 million participants [153], dominated the image of the distribution of participants. The reasons for the American-European gap may be multifaceted. One factor may be the differences in political and administrative frameworks, for example, comparing CE and FDA processes, which may result in slower certification processes for wearables and new technologies in general [31]. Another factor may be cultural mentality resulting in faster adoption of new technology in the United States, as the North Americans own proportionally more fitness trackers in comparison to the Europeans [227,228].

Some factors discussed in other research were not or only briefly mentioned in the included studies [6,29,31], but should also be reflected, especially technical and legal aspects, such as data security [224], data synching, and export. For example, the Germany-based study of Koehler et al [114] was one of the few that detailed data security and transfer of home-based telemonitoring data to the clinic. Data security and privacy are severely governed by the European Union General Data Protection Regulation, which is according to their website the “toughest privacy and security law in the world” [229]. Administrative limitations and challenges presumably obscure the benefits of wearable research in Europe. A possible solution for data security and usability might be data trusts [230] as an alternative to large platforms.

Most medical fields represented in the included studies showed similarities with other reviews [224], for example, studies often focusing on cardiology, sports medicine, and neurology. However, we found a multitude of studies from multidisciplinary fields as well as the field of global health, indicating a likely adoption and expansion of wearables in other medical fields. This underlines the potential for wearables in health research beyond a mere trend or hype, as wearables may provide new possibilities for a broad spectrum of health research, such as for infectious disease prediction like COVID-19 or fertility awareness, among many others.

### Wearable Characteristics

Similar to other reviews, most devices were wrist-worn fitness trackers and accelerometers, and most of them are from the company Fitbit, measuring PA, HR, and sleep [3,27,31,224,225]. These vital signs and device types seem to become the standard in wearable research [3,27,31,224,225]. The included studies also emphasized the growing wearable use [147,195,197], which is also reflected in commercially available devices [38-40]. Currently, further wearable devices emerge, measuring, for example, oximetry, blood pressure, skin temperature, or respiratory rate.

### Categorization of Wearable Application in the Studies

In general, the included studies covered a great scope of health applications such as fertility tracking; monitoring of body characteristics such as weight or diseases such as Alzheimer disease, diabetes mellitus, and AF; as well as associations of coffee intake, sleep, and PA, or blood pressure and steps. We have noted an increase in smaller studies that also included rare populations and conditions, such as fibromyalgia or the rare genetic Pompe disease, indicating that wearables may be valuable for insights into patients with rare conditions. Using affordable, consumer-grade wearables for rare disease assessment and monitoring might eventually be less expensive than specifically developed devices and easier to use for patients. Therefore, currently underrepresented populations may be better researched through wearables [231], that is, different ethnic groups, nationalities, individuals with disabilities, or (rare) conditions. Future studies could examine the participation of underrepresented groups in wearable research in greater depth, particularly in studies analyzing wearable user data.

### Global Health and Low-Resource Contexts

Included studies are predominantly from high-income countries, constituting a gap in wearable studies in low-resource contexts. The AliveCor device was shown to be feasible in Kenya to help detect AF [232], as well as for early diagnosis. The literature underlines the potential for wearable-based research in low-resource settings to generate data and improve health care [9], based on their low cost and ease of use (data acquisition, hardware, and software handling) [233]. Xu et al [234] emphasized that physiological monitoring with wearables hold “promise for substantial improvements in neonatal outcomes” in low- and middle-resource countries. Wearables can generate a solid database for global health research, particularly for morbidity measurements [235], large-scale studies, and modeling and descriptive studies. Topics such as climate change–induced impacts focusing on extreme weather events as an outcome and impact on health [236] may be approached. For example, 1 study [20] measured the physiological response of farm workers to climate conditions with wearables to investigate heat-related illness in a high-income setting. Lam et al [237] investigated the thermal adaptation and comfort of participants originating from various climatic regions. The fitness tracker measured HR data was integrated with other weather and human-based measurements and predicted the thermal sensation of nonlocal participants, among others. Similar studies can be conducted in low-resource regions.

### Strengths and Shortcomings of Wearables

A few studies have experienced issues or shortcomings, such as inaccuracies in measurements and technical issues. Nevertheless, most authors were satisfied with wearables, as strengths were mentioned more frequently than shortcomings. Novelty and innovation outweighed the shortcomings for most authors. The most mentioned positive wearable characteristics were validity and accuracy, technical reliability, innovation, and unobtrusiveness. Only a few authors have mentioned data access through APIs or cloud platforms as a strength. However, the practical value of wearables is heavily reliant on the mode and reliability of data access. Depending on the company, there may be different data access policies in place, whereby it may not be possible to access the raw data of the wearable. Most authors have not considered wearable data access. However, data access and availability of wearable devices is an important aspect that researchers need to be aware of before using a potential study device. Another aspect is open access to the wearables’ raw data or source codes, as companies might change the source code and implement algorithms without the obligation to announce or detail changes that might lead to bias and inconsistency of data [224]. For example, Thijs et al [195] mentioned the consequences of nondisclosed algorithms (Fitbit) for data analysis and standardization. Moreover, the lack of standardization and replicability of wearable raw data and analysis [28] hinders comparability among studies.

Most studies mentioned and discussed validation, accuracy, and certification of the used wearables as part of good research practice approaches. However, the mention of validation or accuracy did not necessarily imply that the wearables had been certified (FDA or CE) or validated in peer-reviewed research. Nevertheless, the authors reported that the wearable device is sufficiently accurate even with existing inaccuracies [14,143,197]. The authors seemed to tolerate smaller inaccuracies and validation drawbacks—especially of established consumer-grade wearables—if usability was of high importance, such as in large-scale studies.

### Large-scale and Big Data Sets for Wearable Research

We noted an increase in large-scale wearable studies in recent years, which is consistent with previous literature [225]. During the COVID-19 pandemic, there has been an increase in studies using secondary data. Studies aimed at generating insights with regard to the developing pandemic, focusing on forecasting models and their effects on different populations. Overall, wearable-generated big data sets might decrease biased data because measurements are objectively taken in the natural environment of numerous and diverse individuals. Although data analytic skills are needed for handling big data sets, their analysis might be extremely valuable for health research in generating new evidence [31,225].

### Limitations

First, not all studies using wearables might have been identified by our search. We included only the wearables of companies in the search that had the highest market share. Therefore, the wearables of smaller or new companies may be missing in this review. In addition, we only included studies published in English, which may have excluded evidence from other regions that may not publish in English. Although this review provides a wide scope of wearable research, the list of included studies is by no means exhaustive.

In addition, wearable costs are only approximations and could be imprecise: (1) companies follow different sales and distribution models, for example membership, rental, and subscription; (2) we only incorporated wearable (hardware) prices, excluding costs for software, maintenance, and other charges such as subscription fees, which may even exceed wearable hardware costs; *and* (3) sales prices are subject to fluctuation. We also excluded many studies as wearables were discontinued. The fluctuant and unstable market, therefore, might also be a factor in decisions regarding the use of wearables [28]. Although interesting and promising, some wearables and similar devices were beyond the scope of this study but might also be valuable for health research. We have provided a wide overview of wearable devices; however, the included studies did not show the full range of possible wearables and measured vital signs [9,37].

In addition, we report the opinions of the included studies with regard to the shortcomings and strengths of wearables. Although these insights might be helpful, they are not objective measures. Moreover, our introduced categories for studies and aims to use wearables might overlap, as separation and categorization are artificial.

### Conclusions

We see a growing uptake of wearables in health research and a trend to use wearables for large-scale, population-based studies. Wearables, which were often piloted in the included studies, were used in diverse health fields including COVID-19 prediction, fertility awareness, geriatrics, AF detection, evaluation of methods, drug effects, psychological interventions, and patient-reported outcomes. Measurement of steps, PA, HR, and sleep may be considered standard wearable measurements. Nevertheless, wearables are becoming more diverse in their measurements and appearance. Therefore, wearable-induced research may include currently underrepresented populations such as the older adults, participants who are disabled, participants with rare chronic or genetic diseases, participants from low socioeconomic backgrounds, and others.

For many researchers, novelty and innovation seem to outweigh shortcomings such as measurement inaccuracies. Overall, the included studies shared key characteristics that the wearables should meet: validity, technical reliability (including data access solutions), innovation, and unobtrusiveness.

We identified a lack of wearable research in low-resource settings. We assume that the reasons for the gap may be a lack of funding and doubts about the usefulness of the wearables. However, wearable devices may be used to generate data in such settings, which may otherwise be difficult and expensive to obtain. Therefore, wearable devices may be valuable for health research in a global context. During the COVID-19-pandemic in particular, large-sized wearable studies were used to generate insights into the developing pandemic and may potentially lead to novel insights into population health trends and forecasts. Future research is needed to determine the usability of wearable devices for underrepresented populations, as well as the feasibility and usefulness of health research in low-resource contexts.

## Appendix

Multimedia Appendix 1 PRISMA-ScR (Preferred Reporting Items for Systematic Reviews and Meta-Analyses extension for Scoping Reviews) checklist.

Multimedia Appendix 2 Details on search and search strings.

Multimedia Appendix 3 Medical Education Research Study Quality Instrument scores of included studies.

Multimedia Appendix 4 Vital signs measured by studies.

Multimedia Appendix 5 Categorization of wearable applications in the studies: article references and examples.

## Abbreviations

AF: atrial fibrillation
API: application programing interface
CE: Communauté Européenne
ECG: electrocardiogram
FDA: Food and Drug Administration
HR: heart rate
PA: physical activity
PRISMA: Preferred Reporting Items for Systematic Reviews and Meta-Analyses
PRISMA-ScR: Preferred Reporting Items for Systematic Reviews and Meta-Analyses extension for Scoping Reviews
