# Supplementary material for: The Impact of Wearable Technologies in Health Research: Scoping Review
Source: JMIR Mhealth Uhealth. 2022 Jan 25;10(1):e34384. doi: 10.2196/34384 (PMC8826148; doi:10.2196/34384)
Supplement: Multimedia Appendix 2 [file mhealth_v10i1e34384_app2.docx]

### Multimedia Appendix 2

#### Details on methods

The extraction form built in the systematic review management platform (Covidence) [44] was tested by the team for 50 references before use. A protocol of this review was not published.

#### Search strings

Last search date was 01.10.2020.

Pubmed:

( ("smartwatc*" OR "smart ring" OR "fitbit" OR "garmin" OR "xiaomi" OR "huawei" OR "withings" OR "vivowatc*" OR "bellabeat" OR "healbe" OR "huami" OR "Amazfit" OR "xiaomi" OR "Airoha" OR "underarmour" OR "omate" OR "zeblaze" OR "oumax" OR "Technaxx" OR "mykronoz" ) OR ( ( "wearabl*" OR "mHealth" OR "pHealth" OR "health track*" OR "fitness track*" OR "fitness watc*" OR "misfit" OR "jawbone" OR "fossil" OR "google watc*" OR "wear OS" OR "apple watc*" OR "watchOS" OR "android wear" OR "nokia" OR "asus" OR "samsung" OR "adidas" OR "lifesense" OR "lifeband" OR "polar" OR "awatch" OR "sony" OR "Motorola" OR "pebble" OR "mi pro fitness" OR "mobile action" OR "wristband measuremen*" OR "medical internet of things" OR wearable electronic devices[MeSH Terms] NOT hearing aids[MeSH Terms] NOT smart glasses[MeSH Terms] ) AND ("wris*" OR "wristband*" OR "hand" OR "arm" OR "finger" OR "ring" OR "chest" OR "torso" ) ) ) AND ( "pulse" OR "heart rate" OR "respiratory rate" OR "heart beat" OR "bpm" OR "ecg" OR "electrocardiogram" OR "Atrial Fibrillation" OR "fitness" OR "walking" OR "physical activity" OR "blood pressure" OR "body temperature" OR "body heat" OR "skin temperature" OR "blood oxygen " OR "oxygen saturation" OR "O2" OR "pulse oxymetr*" OR "capnography" OR "Heart rate variability" OR "hrv" OR "plethysmograp*" OR "photoplethysmogra*" OR "electroencephalogra*" OR "eeg" OR "emg" OR "electromyogra*" OR "gyroscope" OR "electrodermal" OR "acceleromet*" ) AND (2013:2020[pdat])

WOS:

( TS=wearabl*  OR  TS="fitness  track*" OR TS=smartwatch* OR TS=”smart ring*”

OR TS=fitbit OR TS=garmin OR TS=xiaomi OR TS=huami OR TS=amazfit OR TS=huawei OR TS=Samsung OR TS=apple OR TS=”wear os” OR TS=watchos OR TS=”android wear” OR TS=jawbone OR TS=lifesense OR TS=bbk OR TS=fossil OR TS=misfit OR TS=adidas OR TS=withings OR TS=nokia OR TS=asus OR TS=vivowatch OR TS=“google watc*” OR TS=”wear os” OR TS=watchos OR TS=”android wear” OR TS=bellabeat OR TS=diesel OR TS=healbe OR TS=”kate spade” OR TS=LG OR TS=lifeband OR TS=mediaTek OR TS=MTK OR TS=airoha OR TS=”Michael kors” OR TS=mobvoi OR TS=ticwatch OR TS=motorola OR TS=motoact OR TS=movado OR TS=timex OR TS=underarmour OR TS=xiaomi OR TS=lifetrak OR TS=ifit OR TS=pebble OR TS=omate OR TS=zeblaze OR TS=oumax OR TS=”mobile action” OR TS=worldsim OR TS=”mi pro fitness” OR TS=polar OR TS=technaxx OR TS=awatch OR TS=tomtom OR TS=mykronoz)

AND

(TS=wris* OR TS=wristband OR TS=hand OR TS=arm OR TS=ring OR TS=chest OR TS=torso)

AND

( TS=”vital sign*” OR TS="heart  rate" OR TS=bpm OR TS=ecg OR TS=”atrial fibrillation” OR TS=electrocardiogram OR TS="blood pressure" OR TS="body temperature” OR TS="blood oxygen” OR TS=”oxygen saturation" OR TS=O2 OR TS="pulse oxymetr*" OR TS=capnography OR TS="Heart rate variability" OR TS=hrv OR TS=plethysmogra* OR TS=photoplethysmogra* OR TS=gyroscope OR TS=acceleromet*)

Ovid:

1. (*wearable electronic devices/ or *fitness trackers/ or *blood pressure monitoring, ambulatory/ or *electrocardiography, ambulatory/ or *monitoring, ambulatory/).sh.

2. (wearabl* or fitness track* or smartwatc*).ti,ab,kw.

3. (wrist or wristband or arm or ring or chest).ti,ab,kw.

4. 1 or 2

5. 3 and 4

6. limit 5 to yr="2013 – 2020”

Resources: Journals@Ovid Full Text September 25, 2020, Your Journals@Ovid, Books@Ovid September 21, 2020, BIOSIS Previews 1969 to 2010, Ovid MEDLINE(R) and Epub Ahead of Print, In-Process & Other Non-Indexed Citations, Daily and Versions(R) 1946 to September 25, 2020

CINAHL:

( wearable technology or wearable devices or wearable sensors OR wearable technology in healthcare OR “wearabl*” OR (smartwatch) OR "smartwatc*" OR "smart ring" OR "fitbit" OR "garmin" OR "xiaomi" OR "huawei" OR "samsung" OR "jawbone" OR "fossil" OR “misfit” OR “adidas” OR "lifesense" OR "bbk" OR "apple" OR "withings" OR "nokia" OR “asus” OR “vivowatc*” OR “bellabeat” OR “diesel” OR "misfit" OR “adidas” OR "google" OR “wear OS” OR “watchos” OR “android wear” OR “healbe” OR “huami” OR “Amazfit” OR “xiaomi” OR “Kate Spade” OR “LG” OR “lifeband” OR "Mediatek" OR “MTK” OR “MT” OR “Airoha” OR “Michael Kors” “misfit” OR “mobvoi” OR “Ticwatch” OR “Motorola” OR “motoact” OR “Movado” OR “sony” OR “timex” OR “underarmour” OR "lifetrak" OR "ifit" OR "pebble" OR "omate" OR "zeblaze" OR "oumax" OR "mobile action" OR "worldsim" OR "mi pro fitness" OR "polar" OR "Technaxx" OR "awatch" OR "tomtom" OR "mykronoz" OR mHealth OR pHealth OR “health track*” ) AND ( “wris*” OR “wristband” OR "hand" OR "arm" OR "forear*" OR "finger" OR "ring" OR "chest" OR "forehead" ) AND ( pulse OR (heart rate) OR (bpm) OR (ecg) OR (electrocardiogram) OR (respiratory rate) OR (blood pressure) OR ("body temperature") OR (temperature) OR ("fever") OR (heat) OR (blood oxygen saturation) OR (O2) OR (pulse oxymetr*) OR (capnography) OR ("stroke volume") OR "cardiac output" OR "Heart rate variability" OR "hrv" OR (plethysmograp*) OR (photoplethysmograph*) OR (infection) ) Published Date: 20130101-20201231 AND Apply equivalent subjects
