# Supplementary material for: The Impact of Wearable Technologies in Health Research: Scoping Review
Source: JMIR Mhealth Uhealth. 2022 Jan 25;10(1):e34384. doi: 10.2196/34384 (PMC8826148; doi:10.2196/34384)
Supplement: Multimedia Appendix 3 [file mhealth_v10i1e34384_app3.docx]

### Multimedia Appendix 3

**Table 1.** MERSQI scores of included studies

| Study | Score | Adjusted score | Q1* | Q2* | Q3* | Q4* | Q5* | Q6* | Q7* | Q8* | Q9* | Q10* |
| --- | --- | --- | --- | --- | --- | --- | --- | --- | --- | --- | --- | --- |
| Agarwal 2018 ^46^ | 15.50 | 15.50 | 1.5 | 0.5 | 1.5 | 3 | 1 | 1 | 1 | 1 | 2 | 3 |
| Alghamdi 2020 ^47^ | 14.50 | 14.50 | 1.5 | 0.5 | 0.5 | 3 | 1 | 1 | 1 | 1 | 2 | 3 |
| An 2020 ^48^ | 12.00 | 12.80 | 1 | 1.5 | 0.5 | 3 | 0 | 0 | NA | 1 | 2 | 3 |
| Arigo 2020 ^49^ | 11.00 | 13.04 | 1.5 | 0.5 | NA | 3 | NA | 1 | 0 | 1 | 2 | 2 |
| Bade 2018 ^50^ | 13.00 | 14.86 | 1 | 1.5 | 1.5 | 3 | NA | 1 | NA | 1 | 2 | 2 |
| Baril 2019 ^51^ | 10.50 | 14.61 | 1 | 0.5 | NA | 3 | NA | NA | NA | 1 | 2 | 3 |
| Barkley 2019 ^52^ | 10.50 | 12.92 | 1.5 | 0.5 | 0.5 | 3 | NA | NA | NA | 1 | 2 | 2 |
| Barrett 2014 ^53^ | 12.00 | 14.77 | 1.5 | 0.5 | 1 | 3 | NA | NA | NA | 1 | 2 | 3 |
| Bevier 2020 ^54^ | 13.50 | 14.40 | 1.5 | 0.5 | 0.5 | 3 | 1 | 1 | NA | 1 | 2 | 3 |
| Bian 2017 ^55^ | 11.50 | 11.50 | 1 | 0.5 | 0.5 | 3 | 1 | 1 | 0 | 1 | 2 | 1.5 |
| Billeci 2018 ^56^ | 13.50 | 17.28 | 2 | 1.5 | NA | 3 | NA | 1 | NA | 1 | 2 | 3 |
| Birkeland 2017 ^57^ | 15.50 | 16.53 | 3 | 1 | 0.5 | 3 | 1 | 1 | NA | 1 | 2 | 3 |
| Blackshear and Seyfried 2019 ^58^ | 12.50 | 13.79 | 2 | 0.5 | NA | 3 | 0 | 1 | 1 | 1 | 2 | 2 |
| Block 2017 ^60^ | 13.50 | 14.40 | 1.5 | 0.5 | 0.5 | 3 | 1 | 1 | NA | 1 | 2 | 3 |
| Block 2019 ^59^ | 12.50 | 12.50 | 1.5 | 0.5 | 0.5 | 3 | 0 | 1 | 1 | 1 | 2 | 2 |
| Bolourchi and Batra 2015 ^61^ | 14.00 | 14.00 | 1 | 1.5 | 0.5 | 3 | 0 | 1 | 1 | 1 | 2 | 3 |
| Boscolo Alvarez 2017 ^62^ | 11.50 | 13.14 | 2 | 0.5 | 0.5 | 3 | 1 | NA | NA | 1 | 2 | 1.5 |
| Brazendale 2020 ^63^ | 11.50 | 13.14 | 1.5 | 0.5 | 0.5 | 3 | NA | NA | 1 | 1 | 2 | 2 |
| Brudy 2020 ^64^ | 14.00 | 17.23 | 2 | NA | NA | 3 | 1 | 1 | 1 | 1 | 2 | 3 |
| Buchan 2019 ^65^ | 13.00 | 13.00 | 1 | 1.5 | 0.5 | 3 | 0 | 1 | 1 | 1 | 2 | 2 |
| Burnett-Zeigler 2018 ^66^ | 13.50 | 14.90 | 1 | 1.5 | NA | 3 | 1 | 1 | 0 | 1 | 2 | 3 |
| Cai 2019 ^67^ | 14.00 | 15.45 | 1 | 1 | NA | 3 | 1 | 1 | 1 | 1 | 2 | 3 |
| Carrasco 2019 ^68^ | 15.00 | 15.00 | 2 | 0.5 | 0.5 | 3 | 1 | 1 | 1 | 1 | 2 | 3 |
| Cascino 2019 ^69^ | 12.00 | 12.80 | 1 | 0.5 | 0.5 | 3 | 1 | 1 | NA | 1 | 2 | 2 |
| Chang 2020 ^70^ | 11.00 | 12.57 | 1 | 0.5 | 0.5 | 3 | NA | 1 | NA | 1 | 2 | 2 |
| Chapple 2020 ^71^ | 13.00 | 15.41 | 1.5 | 1.5 | NA | 3 | NA | 1 | 0 | 1 | 2 | 3 |
| Chue 2018 ^72^ | 12.00 | 12.80 | 1 | 0.5 | 0.5 | 3 | NA | 1 | 0 | 1 | 2 | 3 |
| Cohen-Holzer 2017 ^73^ | 14.50 | 14.50 | 1.5 | 0.5 | 0.5 | 3 | 1 | 1 | 1 | 1 | 2 | 3 |
| Cole 2019 ^74^ | 11.50 | 11.50 | 1.5 | 0.5 | 0.5 | 3 | 0 | 0 | 0 | 1 | 2 | 3 |
| Collier 2020 ^75^ | 12.00 | 13.71 | 1 | 0.5 | 0.5 | 3 | NA | 1 | NA | 1 | 2 | 3 |
| Costa 2019 ^76^ | 11.50 | 14.72 | 1 | 0.5 | NA | 3 | NA | 1 | NA | 1 | 2 | 3 |
| Culp and Tonelli 2019 ^20^ | 13.00 | 13.00 | 1 | 1 | 1 | 3 | 0 | 1 | 0 | 1 | 2 | 3 |
| Currow 2017 ^77^ | 14.50 | 17.19 | 3 | 1.5 | NA | 3 | 1 | 0 | NA | 1 | 2 | 3 |
| DasMahapatra 2018 ^78^ | 12.50 | 13.79 | 1 | 1.5 | NA | 3 | 0 | 1 | 0 | 1 | 2 | 3 |
| Do 2020 ^79^ | 12.50 | 12.50 | 1.5 | 0.5 | 0.5 | 3 | 0 | 1 | 0 | 1 | 2 | 3 |
| Downey 2018 ^80^ | 11.50 | 14.72 | 3 | 0.5 | NA | 3 | NA | 1 | NA | 1 | 0 | 3 |
| Driesman 2020 ^81^ | 11.50 | 13.63 | 1 | 0.5 | NA | 3 | 1 | 1 | NA | 1 | 2 | 2 |
| Elmagboul 2020 ^82^ | 13.50 | 14.40 | 1 | 1 | 0.5 | 3 | 1 | 1 | NA | 1 | 2 | 3 |
| English 2016 ^83^ | 14.50 | 16.00 | 2 | 1.5 | NA | 3 | 1 | 1 | 1 | 1 | 2 | 2 |
| Eyre 2015 ^84^ | 11.50 | 13.63 | 1 | 1.5 | NA | 3 | 0 | 1 | NA | 1 | 2 | 2 |
| Fagherazzi 2017 ^85^ | 13.50 | 16.00 | 1 | 1.5 | NA | 3 | NA | 1 | 1 | 1 | 2 | 3 |
| Fairclough 2019 ^86^ | 14.50 | 16.00 | 1 | 1.5 | NA | 3 | 1 | 1 | 1 | 1 | 2 | 3 |
| Faust 2020 ^87^ | 11.50 | 13.63 | 1 | 0.5 | NA | 3 | 1 | NA | 1 | 1 | 2 | 2 |
| Flatt and Esco 2016 ^88^ | 12.50 | 14.81 | 1 | 0.5 | NA | 3 | 1 | 1 | NA | 1 | 2 | 3 |
| Frie 2020 ^89^ | 13.50 | 14.90 | 1 | 1.5 | NA | 3 | 0 | 1 | 1 | 1 | 2 | 3 |
| Ghomrawi 2018 ^90^ | 13.50 | 13.50 | 1.5 | 0.5 | 0.5 | 3 | 1 | 1 | 0 | 1 | 2 | 3 |
| Girard 2020 ^91^ | 10.50 | 12.44 | 1 | 1.5 | NA | 3 | 0 | 0 | NA | 1 | 2 | 2 |
| Gluck 2020 ^92^ | 14.50 | 14.50 | 1.5 | 0.5 | 0.5 | 3 | 1 | 1 | 1 | 1 | 2 | 3 |
| Green 2019 ^93^ | 12.50 | 13.33 | 1.5 | 0.5 | 0.5 | 3 | 0 | 1 | NA | 1 | 2 | 3 |
| Gresham 2018 ^94^ | 14.00 | 14.00 | 1 | 0.5 | 0.5 | 3 | 1 | 1 | 1 | 1 | 2 | 3 |
| Grimes 2019 ^95^ | 14.50 | 14.50 | 1.5 | 0.5 | 1.5 | 3 | 1 | 1 | 0 | 1 | 2 | 3 |
| Groat 2018 ^96^ | 13.50 | 14.40 | 1.5 | 0.5 | 0.5 | 3 | NA | 1 | 1 | 1 | 2 | 3 |
| Guo 2019 ^98^ | 13.50 | 16.00 | 1 | 1.5 | NA | 3 | NA | 1 | 1 | 1 | 2 | 3 |
| Guo 2020 ^97^ | 14.50 | 16.00 | 1 | 1.5 | NA | 3 | 1 | 1 | 1 | 1 | 2 | 3 |
| Hamed 2019 ^99^ | 13.00 | 14.34 | 1 | 1 | NA | 3 | 0 | 1 | 1 | 1 | 2 | 3 |
| Han 2018 ^100^ | 12.50 | 13.33 | 2 | 1 | 0.5 | 3 | 1 | 0 | NA | 1 | 2 | 2 |
| Hemphill 2020 ^101^ | 14.00 | 14.00 | 1 | 1.5 | 0.5 | 3 | 1 | 1 | 0 | 1 | 2 | 3 |
| Hirschberg 2020 ^102^ | 11.00 | 13.04 | 1.5 | NA | 0.5 | 3 | 0 | 1 | NA | 1 | 2 | 2 |
| Huberty 2016 ^103^ | 12.50 | 13.79 | 1 | 1.5 | NA | 3 | 0 | 1 | 0 | 1 | 2 | 3 |
| Jeong 2019 ^104^ | 13.00 | 14.86 | 1 | 1.5 | 0.5 | 3 | NA | 1 | NA | 1 | 2 | 3 |
| Jiang 2019 ^105^ | 12.00 | 14.22 | 1.5 | 0.5 | NA | 3 | 1 | 1 | NA | 1 | 2 | 2 |
| John-Henderson 2019 ^106^ | 12.50 | 14.81 | 1 | 0.5 | NA | 3 | 1 | 1 | NA | 1 | 2 | 3 |
| Jones 2020 ^107^ | 15.00 | 15.00 | 1 | 1.5 | 0.5 | 3 | 1 | 1 | 1 | 1 | 2 | 3 |
| Jonker 2020 ^108^ | 14.50 | 15.47 | 1.5 | 0.5 | 1.5 | 3 | NA | 1 | 1 | 1 | 2 | 3 |
| Kabbach 2017 ^109^ | 13.50 | 14.40 | 2 | 1 | 0.5 | 3 | NA | 1 | 0 | 1 | 2 | 3 |
| Kagamiyama and Yano 2018 ^110^ | 11.50 | 12.27 | 1 | 0.5 | 1 | 3 | 0 | 0 | NA | 1 | 2 | 3 |
| Kaura 2019 ^111^ | 16.00 | 16.00 | 3 | 1.5 | 0.5 | 3 | 0 | 1 | 1 | 1 | 2 | 3 |
| Kim 2019 ^113^ | 12.50 | 12.50 | 1.5 | 0.5 | 0.5 | 3 | 0 | 1 | 0 | 1 | 2 | 3 |
| Kim 2019 ^112^ | 14.00 | 15.45 | 1.5 | 1.5 | NA | 3 | 1 | 1 | 1 | 1 | 2 | 2 |
| Kimura 2020 ^114^ | 13.50 | 16.00 | 1 | 1.5 | NA | 3 | 1 | 1 | NA | 1 | 2 | 3 |
| Kochiya 2017 ^115^ | 12.00 | 12.00 | 1 | 0.5 | 0.5 | 3 | 1 | 0 | 0 | 1 | 2 | 3 |
| Koehler 2018 ^116^ | 16.50 | 16.50 | 3 | 1.5 | 1 | 3 | 1 | 1 | 0 | 1 | 2 | 3 |
| Kolk 2020 ^117^ | 14.00 | 14.00 | 1 | 1.5 | 1.5 | 3 | 0 | 1 | 1 | 1 | 2 | 2 |
| Kruizinga 2020 ^118^ | 14.50 | 14.50 | 2 | 0.5 | 1 | 3 | 1 | 1 | 0 | 1 | 2 | 3 |
| Lamar 2016 ^119^ | 12.50 | 12.50 | 2 | 0.5 | 1 | 3 | 0 | 1 | 0 | 1 | 2 | 2 |
| Larsen 2020 ^120^ | 13.00 | 16.64 | 1.5 | 1.5 | NA | 3 | NA | 1 | NA | 1 | 2 | 3 |
| Lazaridou 2020 ^121^ | 14.00 | 14.00 | 1 | 0.5 | 1.5 | 3 | 1 | 1 | 0 | 1 | 2 | 3 |
| Lee 2018 ^123^ | 14.50 | 16.00 | 2 | 0.5 | NA | 3 | 1 | 1 | 1 | 1 | 2 | 3 |
| Lee 2019 ^125^ | 12.00 | 14.22 | 1.5 | 0.5 | NA | 3 | 1 | 1 | NA | 1 | 2 | 2 |
| Lee 2020 ^124^ | 14.00 | 14.00 | 2 | 0.5 | 0.5 | 3 | 0 | 1 | 1 | 1 | 2 | 3 |
| LeHello 2018 ^122^ | 11.50 | 14.72 | 1 | 1.5 | NA | 3 | NA | 0 | NA | 1 | 2 | 3 |
| Liao 2020 ^126^ | 13.00 | 13.87 | 1 | 0.5 | 0.5 | 3 | 1 | 1 | NA | 1 | 2 | 3 |
| Lim 2018 ^127^ | 12.50 | 13.79 | 1 | NA | 1.5 | 3 | 0 | 0 | 1 | 1 | 2 | 3 |
| Lim 2018 ^128^ | 12.00 | 14.77 | 1 | NA | NA | 3 | 0 | 1 | 1 | 1 | 2 | 3 |
| Lin 2020 ^129^ | 13.50 | 14.90 | 1 | 1.5 | NA | 3 | 1 | 1 | 0 | 1 | 2 | 3 |
| Liu 2016 ^130^ | 13.00 | 15.41 | 1 | NA | 1 | 3 | NA | 1 | 1 | 1 | 2 | 3 |
| Low 2017 ^132^ | 12.00 | 13.24 | 1.5 | NA | 0.5 | 3 | 1 | 0 | 0 | 1 | 2 | 3 |
| Low 2018 ^131^ | 13.50 | 13.50 | 1.5 | 0.5 | 0.5 | 3 | 1 | 1 | 0 | 1 | 2 | 3 |
| Ma 2018 ^133^ | 14.00 | 14.93 | 2 | 0.5 | 0.5 | 3 | NA | 1 | 1 | 1 | 2 | 3 |
| Maijala 2019 ^134^ | 10.50 | 14.61 | 1 | NA | 0.5 | 3 | NA | NA | NA | 1 | 2 | 3 |
| Makic 2020 ^135^ | 14.00 | 14.93 | 1 | 0.5 | 1.5 | 3 | 1 | 1 | NA | 1 | 2 | 3 |
| Massey 2018 ^137^ | 13.50 | 14.90 | 1 | NA | 0.5 | 3 | 1 | 1 | 1 | 1 | 2 | 3 |
| Massey 2020 ^136^ | 13.50 | 14.90 | 1 | 1.5 | NA | 3 | 1 | 1 | 1 | 1 | 2 | 2 |
| Matcham 2019 ^138^ | 13.00 | 15.41 | 1.5 | 1.5 | NA | 3 | 0 | 1 | NA | 1 | 2 | 3 |
| McLean 2018 ^139^ | 12.50 | 14.81 | 1 | 0.5 | NA | 3 | NA | 1 | 1 | 1 | 2 | 3 |
| Mead 2019 ^140^ | 12.00 | 12.80 | 1 | 0.5 | 0.5 | 3 | NA | 1 | 0 | 1 | 2 | 3 |
| Meijer 2014 ^141^ | 13.00 | 13.87 | 2 | 0.5 | 0.5 | 3 | 1 | 0 | NA | 1 | 2 | 3 |
| Melin 2016 ^142^ | 14.50 | 14.50 | 1.5 | 0.5 | 0.5 | 3 | 1 | 1 | 1 | 1 | 2 | 3 |
| Menai 2017 ^143^ | 14.00 | 16.59 | 1.5 | 1.5 | NA | 3 | NA | 1 | 1 | 1 | 2 | 3 |
| Mendelsohn 2019 ^144^ | 14.00 | 14.93 | 2 | 0.5 | 0.5 | 3 | 1 | 1 | NA | 1 | 2 | 3 |
| Mičková 2019 ^145^ | 13.00 | 13.00 | 2 | 0.5 | 0.5 | 3 | 0 | 1 | 0 | 1 | 2 | 3 |
| Miller 2020 ^146^ | 14.00 | 16.59 | 1.5 | 1.5 | NA | 3 | 1 | NA | 1 | 1 | 2 | 3 |
| Mobbs 2016 ^147^ | 13.50 | 13.50 | 1.5 | 0.5 | 0.5 | 3 | 1 | 1 | 0 | 1 | 2 | 3 |
| Mocny-Pachońska 2020 ^148^ | 14.50 | 16.00 | 3 | 0.5 | NA | 3 | 1 | 1 | 0 | 1 | 2 | 3 |
| Modarress-Sadeghi 2019 ^149^ | 15.50 | 15.50 | 1 | 1.5 | 1 | 3 | 1 | 1 | 1 | 1 | 2 | 3 |
| Mora-Gonzalez 2019 ^150^ | 14.50 | 16.00 | 1 | NA | 1.5 | 3 | 1 | 1 | 1 | 1 | 2 | 3 |
| Morcos 2020 ^151^ | 13.00 | 13.87 | 1 | 0.5 | 0.5 | 3 | 1 | 1 | NA | 1 | 2 | 3 |
| Morhardt 2017 ^152^ | 14.00 | 14.00 | 1 | 0.5 | 0.5 | 3 | 1 | 1 | 1 | 1 | 2 | 3 |
| Mueller 2019 ^153^ | 14.00 | 16.59 | 1.5 | 1.5 | NA | 3 | 1 | 1 | NA | 1 | 2 | 3 |
| Murphree 2017 ^154^ | 14.50 | 17.19 | 3 | 0.5 | NA | 3 | 1 | 1 | NA | 1 | 2 | 3 |
| Natarajan 2019 ^155^ | 12.50 | 13.79 | 1 | 1.5 | NA | 3 | 1 | 0 | 0 | 1 | 2 | 3 |
| Niemela 2019 ^156^ | 14.50 | 16.00 | 1 | 1.5 | NA | 3 | 1 | 1 | 1 | 1 | 2 | 3 |
| Nishida 2017 ^157^ | 12.00 | 12.80 | 1 | 0.5 | 0.5 | 3 | NA | 1 | 0 | 1 | 2 | 3 |
| Nowell 2019 ^158^ | 14.00 | 14.00 | 1 | 1.5 | 0.5 | 3 | 1 | 1 | 0 | 1 | 2 | 3 |
| Orme 2019 ^159^ | 14.00 | 16.00 | 2 | 1.5 | 0.5 | 3 | NA | 1 | NA | 1 | 2 | 3 |
| Otsuki und Ishii 2017 ^160^ | 13.50 | 16.00 | 1 | 1.5 | NA | 3 | NA | 1 | 1 | 1 | 2 | 3 |
| Pakhomov 2020 ^161^ | 14.00 | 14.93 | 1.5 | 0.5 | 1 | 3 | NA | 1 | 1 | 1 | 2 | 3 |
| Pastor 2020 ^162^ | 12.00 | 13.24 | 2 | 1 | NA | 3 | 0 | 0 | 0 | 1 | 2 | 3 |
| Peach 2018 ^163^ | 13.00 | 14.34 | 1 | 1 | NA | 3 | 1 | 1 | 0 | 1 | 2 | 3 |
| Pépin 2020 ^164^ | 10.50 | 11.59 | 1 | 1.5 | NA | 3 | 1 | 1 | 0 | 1 | 0 | 2 |
| Perez 2019 ^15^ | 11.50 | 13.63 | 1 | 1.5 | NA | 3 | 0 | 0 | NA | 1 | 2 | 3 |
| Phillips 2018 ^165^ | 14.50 | 14.50 | 1 | 1.5 | 1 | 3 | 1 | 1 | 0 | 1 | 2 | 3 |
| Pozehl 2018 ^166^ | 13.50 | 14.40 | 1 | 1 | 0.5 | 3 | 1 | 1 | NA | 1 | 2 | 3 |
| Pradhan and Kelly 2019 ^167^ | 15.50 | 17.10 | 2 | 1.5 | NA | 3 | 1 | 1 | 1 | 1 | 2 | 3 |
| Quer 2020 ^168^ | 14.50 | 16.00 | 1 | 1.5 | NA | 3 | 1 | 1 | 1 | 1 | 2 | 3 |
| Quer 2020 ^169^ | 13.50 | 16.00 | 1 | 1.5 | NA | 3 | NA | 1 | 1 | 1 | 2 | 3 |
| Quiroz 2018 ^170^ | 12.50 | 14.81 | 1 | 0.5 | NA | 3 | 1 | 1 | NA | 1 | 2 | 3 |
| Radin 2020 ^14^ | 13.50 | 16.00 | 1 | 1.5 | NA | 3 | NA | 1 | 1 | 1 | 2 | 3 |
| Rahman 2020 ^172^ | 12.50 | 12.50 | 2 | 1 | 0.5 | 3 | 0 | 0 | 0 | 1 | 2 | 3 |
| Rahman 2020 ^171^ | 12.00 | 12.80 | 1 | 0.5 | 0.5 | 3 | 1 | 0 | NA | 1 | 2 | 3 |
| Ramirez 2020 ^173^ | 15.50 | 17.10 | 2 | 1.5 | NA | 3 | 1 | 1 | 1 | 1 | 2 | 3 |
| Raywood 2020 ^174^ | 12.50 | 13.33 | 1.5 | 1.5 | 0.5 | 3 | 0 | 0 | NA | 1 | 2 | 3 |
| Reddy 2018 ^175^ | 13.00 | 13.00 | 3 | 0.5 | 0.5 | 3 | 0 | 0 | 0 | 1 | 2 | 3 |
| Roe 2016 ^176^ | 14.00 | 15.45 | 1.5 | NA | 0.5 | 3 | 1 | 1 | 1 | 1 | 2 | 3 |
| Root 2017 ^177^ | 13.50 | 16.00 | 1 | 1.5 | NA | 3 | 1 | 1 | NA | 1 | 2 | 3 |
| Rykov 2020 ^178^ | 14.50 | 16.00 | 1 | 1.5 | NA | 3 | 1 | 1 | 1 | 1 | 2 | 3 |
| Saarikko 2020 ^179^ | 16.50 | 16.50 | 1.5 | 1.5 | 1.5 | 3 | 1 | 1 | 1 | 1 | 2 | 3 |
| Saif 2020 ^180^ | 14.50 | 14.50 | 1 | 1 | 1.5 | 3 | 1 | 1 | 0 | 1 | 2 | 3 |
| Scarlett 2020 ^181^ | 12.50 | 14.81 | 1 | 1.5 | NA | 3 | 1 | 0 | NA | 1 | 2 | 3 |
| Sehgal 2019 ^182^ | 13.50 | 14.90 | 1 | 0.5 | NA | 3 | 1 | 1 | 1 | 1 | 2 | 3 |
| Semaan 2020 ^183^ | 15.50 | 17.10 | 2 | 1.5 | NA | 3 | 1 | 1 | 1 | 1 | 2 | 3 |
| Shaw 2019 ^184^ | 12.50 | 13.79 | 1 | 0.5 | NA | 3 | 1 | 1 | 0 | 1 | 2 | 3 |
| Shen 2017 ^185^ | 13.00 | 13.87 | 2 | 0.5 | 0.5 | 3 | 1 | 0 | NA | 1 | 2 | 3 |
| Shilaih 2017 ^187^ | 14.00 | 14.00 | 1 | 0.5 | 0.5 | 3 | 1 | 1 | 1 | 1 | 2 | 3 |
| Shilaih 2018 ^186^ | 13.50 | 13.50 | 1.5 | 0.5 | 0.5 | 3 | 1 | 1 | 0 | 1 | 2 | 3 |
| Shufelt 2020 ^188^ | 11.50 | 13.63 | 1 | NA | 1.5 | 3 | 0 | 0 | NA | 1 | 2 | 3 |
| Silverman-Lloyd 2018 ^189^ | 13.50 | 16.00 | 1 | 1.5 | NA | 3 | 1 | 1 | NA | 1 | 2 | 3 |
| Smith 2016 ^190^ | 12.50 | 14.81 | 1 | 0.5 | NA | 3 | 1 | 1 | NA | 1 | 2 | 3 |
| Solomon 2016 ^191^ | 11.50 | 13.63 | 1 | 0.5 | NA | 3 | 1 | 0 | NA | 1 | 2 | 3 |
| Souza 2019 ^192^ | 15.00 | 16.55 | 1.5 | 1.5 | NA | 3 | 1 | 1 | 1 | 1 | 2 | 3 |
| Steinhubl 2018 ^193^ | 16.00 | 16.00 | 3 | 1.5 | 0.5 | 3 | 1 | 1 | 0 | 1 | 2 | 3 |
| Stelzer 2018 ^194^ | 13.00 | 16.00 | 3 | NA | NA | 3 | 0 | 1 | 0 | 1 | 2 | 3 |
| Strain 2020 ^195^ | 12.50 | 14.81 | 1 | 1.5 | NA | 3 | 0 | 1 | NA | 1 | 2 | 3 |
| Tateishi 2014 ^196^ | 14.50 | 16.00 | 3 | 0.5 | NA | 3 | 1 | 1 | 0 | 1 | 2 | 3 |
| Thijs 2019 ^197^ | 14.00 | 14.00 | 2 | 0.5 | 0.5 | 3 | 1 | 1 | 0 | 1 | 2 | 3 |
| Thomas 2016 ^198^ | 11.50 | 12.69 | 1 | 1.5 | NA | 3 | 0 | 0 | 0 | 1 | 2 | 3 |
| Thompson 2018 ^199^ | 15.50 | 17.10 | 2 | 1.5 | NA | 3 | 1 | 1 | 1 | 1 | 2 | 3 |
| Thorup 2016 ^200^ | 16.50 | 16.50 | 3 | 1 | 0.5 | 3 | 1 | 1 | 1 | 1 | 2 | 3 |
| Tomitani 2020 ^201^ | 13.00 | 13.87 | 1 | 0.5 | 0.5 | 3 | 1 | 1 | NA | 1 | 2 | 3 |
| Turakhia 2015 ^202^ | 14.00 | 14.00 | 1 | 0.5 | 0.5 | 3 | 1 | 1 | 1 | 1 | 2 | 3 |
| Turel 2016 ^203^ | 12.50 | 13.33 | 1 | 1 | 0.5 | 3 | 1 | 1 | NA | 1 | 2 | 2 |
| Twiggs 2018 ^204^ | 14.50 | 14.50 | 1.5 | 0.5 | 0.5 | 3 | 1 | 1 | 1 | 1 | 2 | 3 |
| Van der Kamp 2019 ^205^ | 11.00 | 11.73 | 1 | 0.5 | 0.5 | 3 | 0 | 0 | NA | 1 | 2 | 3 |
| Van Wamelen 2019 ^206^ | 13.50 | 14.90 | 1 | 0.5 | NA | 3 | 1 | 1 | 1 | 1 | 2 | 3 |
| Vaughn 2019 ^207^ | 13.50 | 13.50 | 1.5 | 0.5 | 0.5 | 3 | 1 | 1 | 0 | 1 | 2 | 3 |
| Venkataramanan 2019 ^208^ | 13.50 | 14.90 | 1 | 0.5 | NA | 3 | 1 | 1 | 1 | 1 | 2 | 3 |
| Wallace 2016 ^209^ | 15.00 | 15.00 | 2 | 0.5 | 0.5 | 3 | 1 | 1 | 1 | 1 | 2 | 3 |
| Wang 2020 ^210^ | 10.50 | 12.44 | 1 | 0.5 | NA | 3 | 0 | 0 | NA | 1 | 2 | 3 |
| Weatherall 2018 ^211^ | 12.50 | 13.79 | 1 | 0.5 | NA | 3 | 0 | 1 | 1 | 1 | 2 | 3 |
| Weeks 2018 ^212^ | 14.00 | 14.00 | 1 | 0.5 | 0.5 | 3 | 1 | 1 | 1 | 1 | 2 | 3 |
| Wiles 2020 ^213^ | 11.50 | 13.63 | 1 | 1.5 | NA | 3 | 0 | 0 | NA | 1 | 2 | 3 |
| Winnebeck 2018 ^214^ | 13.50 | 14.90 | 1 | 1.5 | NA | 3 | 1 | 1 | 0 | 1 | 2 | 3 |
| Wyatt 2020 ^215^ | 11.50 | 16.00 | 1 | 1.5 | NA | 3 | NA | NA | NA | 1 | 2 | 3 |
| Xu 2018 ^216^ | 13.50 | 16.00 | 1 | 1.5 | NA | 3 | 1 | 1 | NA | 1 | 2 | 3 |
| Yang 2019 ^217^ | 11.00 | 11.73 | 1 | 0.5 | 0.5 | 3 | 0 | 0 | NA | 1 | 2 | 3 |
| Youn and Lee 2020 ^218^ | 12.00 | 12.00 | 1 | 0.5 | 0.5 | 3 | 1 | 1 | 0 | 1 | 2 | 2 |
| Yu 2020 ^219^ | 11.50 | 13.63 | 1 | 0.5 | NA | 3 | 1 | 1 | NA | 1 | 2 | 2 |
| Zhu 2019 ^220^ | 13.50 | 14.90 | 1 | 1.5 | NA | 3 | 0 | 1 | 1 | 1 | 2 | 3 |
| Zhuo 2020 ^221^ | 14.50 | 14.50 | 1 | 1.5 | 1 | 3 | 0 | 1 | 1 | 1 | 2 | 3 |

* Questions 1 – 10 of the MERSQI scale

For all included studies, the MERSQI scores are listed, i.e., sums of the ten questions, as well as adjusted scores, i.e., sums adjusted for not applicable and therefore missing values.
