# Supplementary material for: The Impact of Wearable Technologies in Health Research: Scoping Review
Source: JMIR Mhealth Uhealth. 2022 Jan 25;10(1):e34384. doi: 10.2196/34384 (PMC8826148; doi:10.2196/34384)
Supplement: Multimedia Appendix 4 [file mhealth_v10i1e34384_app4.docx]

### Multimedia Appendix 4

**Table 2.** Vital signs measured by studies

| **Study authors,**  **Year of Publication** | **Cardiac** | | | **Physical activity (PA)** | | | | | | **Other** | | | | | | **Wearables** |
| --- | --- | --- | --- | --- | --- | --- | --- | --- | --- | --- | --- | --- | --- | --- | --- | --- |
|  | **Heart rate** | **Heart rate variability** | **ECG (/heart rythm analysis)** | **PA - steps** | **PA - intensity (MVPA)** | **PA - energy expenditure (calories)** | **PA - raw, axial movements** | **PA - distance** | **PA - other** | **Sleep - time (onset, etc.)** | **Sleep - quality** | **Blood / pulse pressure** | **Skin temperature** | **Respiratory rate** | **Oximetry** |  |
| Agarwal 2018 [46] |  |  |  | **x** |  |  |  |  |  | **x** | **x** |  |  |  |  | Fitbit Charge |
| Alghamdi 2020 [47] |  |  |  | **x** | **x** |  |  |  |  | **x** |  |  |  |  |  | Fitbit Flex |
| An 2020 [48] |  |  |  | **x** | **x** | **x** |  |  | **x** |  |  |  |  |  |  | Fitbit Charge |
| Arigo 2020 [49] |  |  |  | **x** | **x** |  |  |  |  |  |  |  |  |  |  | Fitbit Flex |
| Bade 2018 [50] |  |  |  | **x** |  |  |  |  |  |  |  |  |  |  |  | Fitbit Zip |
| Baril 2019 [51] |  |  |  | **x** |  |  |  |  |  |  |  |  |  |  |  | Fitbit Flex |
| Barkley 2019 [52] |  |  |  | **x** |  |  |  |  |  |  |  |  |  |  |  | Fitbit Zip |
| Barrett 2014 [53] |  |  |  |  |  |  |  |  |  |  |  |  |  |  |  | Zio Patch |
| Bevier 2020 [54] |  |  |  | **x** |  | **x** |  |  |  |  |  |  |  |  |  | Fitbit Charge, ActiGraph wGT3X-BT |
| Bian 2017 [55] |  |  |  |  |  |  |  |  | **x** |  | **x** |  |  |  |  | Fitbit Charge |
| Billeci 2018 [56] |  | **x** |  |  |  |  |  |  |  |  |  |  |  |  |  | Shimmer ECG (modified with Polar chest strap) |
| Birkeland 2017 [57] |  |  |  | **x** |  |  |  |  |  |  |  |  |  |  |  | Fitbit Flex |
| Blackshear and Seyfried 2019 [58] |  |  |  | **x** |  |  |  |  |  |  |  |  |  |  |  | Fitbit Zip |
| Block 2017 [60] |  |  |  | **x** |  |  |  |  |  |  |  |  |  |  |  | Fitbit Flex, ActiGraph GT3X |
| Block 2019 [59] |  |  |  | **x** |  |  |  |  |  |  |  |  |  |  |  | Fitbit Flex |
| Bolourchi and Batra 2015 [61] |  |  | **x** |  |  |  |  |  |  |  |  |  |  |  |  | Zio Patch |
| Boscolo Alvarez 2017 [62] |  | **x** | **x** |  |  |  |  |  |  |  |  |  |  |  |  | Polar chest-strap (+ receiver) |
| Brazendale 2020 [63] | **x** |  |  | **x** | **x** |  |  |  |  | **x** |  |  |  |  |  | Fitbit Alta |
| Brudy 2020 [64] |  |  |  | **x** | **x** |  |  |  |  |  |  |  |  |  |  | Garmin Vivofit |
| Buchan 2019 [65] |  |  |  |  | **x** |  | **x** |  |  |  |  |  |  |  |  | ActiGraph wGT3X-BT |
| Burnett-Zeigler 2018 [66] | **x** |  |  |  |  |  |  |  |  |  |  | **x** |  |  |  | Zensorium activity tracker |
| Cai 2019 [67] |  |  |  | **x** |  |  |  |  |  |  |  |  |  |  |  | Fitbit devices |
| Carrasco 2019 [68] |  |  |  | **x** |  | **x** |  |  | **x** |  |  |  |  |  |  | Fitbit Charge |
| Cascino 2019 [69] |  |  |  | **x** |  |  |  |  |  |  |  |  |  |  |  | Fitbit Zip |
| Chang 2020 [70] |  |  |  | **x** |  | **x** |  | **x** |  |  |  |  |  |  |  | Xiaomi Mi Band |
| Chapple 2020 [71] |  |  |  |  |  |  |  |  |  | **x** | **x** |  |  |  |  | Withings Steel, Withings Pulse Ox tracker |
| Chue 2018 [72] |  |  |  |  |  |  |  |  |  | **x** | **x** |  |  |  |  | Fitbit Flex |
| Cohen-Holzer 2017 [73] | **x** | **x** |  |  |  |  |  |  |  |  |  |  |  |  |  | Polar chest-strap (+ receiver) |
| Cole 2019 [74] | **x** | **x** |  | **x** |  | **x** |  | **x** |  |  |  |  |  |  | **x** | Wavelet wristband (now: Biostrap) |
| Collier 2020 [75] |  | **x** |  |  |  |  |  |  |  |  | **x** |  |  |  |  | Fitbit Charge |
| Costa 2019 [76] | **x** |  | **x** |  |  |  |  |  |  |  | **x** |  |  |  |  | Firstbeat Bodyguard2, Actigraph wGT3X-BT |
| Culp and Tonelli 2019 [20] | **x** |  |  |  | **x** | **x** |  |  |  |  |  |  |  |  |  | Zephyr status monitor |
| Currow 2017 [77] |  |  |  | **x** |  |  |  |  |  | **x** |  |  |  |  |  | Fitbit Charge |
| DasMahapatra 2018 [78] |  |  |  | **x** |  |  |  |  |  |  |  |  |  |  |  | Fitbit One |
| Do 2020 [79] |  |  |  | **x** |  |  |  |  |  | **x** |  |  |  |  |  | Fitbit Flex |
| Downey 2018 [80] | **x** |  |  |  |  |  |  |  |  |  |  |  | **x** | **x** |  | SensiumVitals patch |
| Driesman 2020 [81] |  |  |  | **x** |  |  |  |  |  |  |  |  |  |  |  | Fitbit Flex |
| Elmagboul 2020 [82] | **x** |  |  | **x** |  |  |  |  |  | **x** |  |  |  |  |  | Fitbit Charge |
| English 2016 [83] |  |  |  |  | **x** |  | **x** |  |  |  |  |  |  |  |  | ActiGraph GT3X+ |
| Eyre 2015 [84] | **x** |  |  |  |  |  |  |  |  |  |  |  |  |  |  | Garmin Forerunner |
| Fagherazzi 2017 [85] | **x** |  |  | **x** |  |  |  |  |  | **x** | **x** |  |  |  |  | Withings trackers |
| Fairclough 2019 [86] |  |  |  |  |  |  | **x** |  |  |  |  |  |  |  |  | ActiGraph GT9X |
| Faust 2020 [87] | **x** |  |  |  | **x** |  |  |  |  | **x** |  |  |  |  |  | Fitbit Charge |
| Flatt and Esco 2016 [88] |  | **x** |  |  |  |  |  |  |  |  |  |  |  |  |  | Polar chest-strap (+ receiver) |
| Frie 2020 [89] |  |  |  | **x** |  |  |  |  |  |  |  |  |  |  |  | Withings trackers |
| Ghomrawi 2018 [90] |  |  |  |  | **x** |  |  |  |  |  |  |  |  |  |  | ActiGraph GT3X / wGT3X-BT |
| Girard 2020 [91] |  |  |  | **x** | **x** |  |  |  |  |  |  |  |  |  |  | Fitbit Flex |
| Gluck 2020 [92] |  |  |  | **x** |  |  |  |  |  |  |  |  |  |  |  | Fitbit Flex, One |
| Green 2019 [93] | **x** |  |  | **x** |  | **x** |  |  |  | **x** |  |  |  |  |  | Fitbit Charge |
| Gresham 2018 [94] | **x** |  |  | **x** |  |  |  | **x** | **x** |  |  |  |  |  |  | Fitbit Charge |
| Grimes 2019 [95] |  |  |  |  |  |  | **x** |  |  |  |  |  |  |  |  | Axivity AX3 |
| Groat 2018 [96] | **x** |  |  |  |  |  |  |  | **x** |  |  |  |  |  |  | Fitbit Charge |
| Guo 2019 [98] | **x** |  | **x** |  |  |  |  |  |  |  |  |  |  |  |  | Huawei smartwatches and trackers |
| Guo 2020 [97] | **x** |  | **x** |  |  |  |  |  |  |  |  |  |  |  |  | Huawei smartwatches and trackers |
| Hamed 2019 [99] |  |  |  | **x** |  |  |  |  | **x** | **x** |  |  |  |  |  | Fitbit One |
| Han 2018 [100] |  |  |  | **x** |  |  |  | **x** |  |  |  |  |  |  |  | Fitbit Flex |
| Hemphill 2020 [101] |  |  |  | **x** |  |  |  |  |  |  |  |  |  |  |  | Fitbit Charge |
| Hirschberg 2020 [102] | **x** |  |  | **x** |  |  |  |  |  | **x** |  |  |  |  |  | Fitbit Charge |
| Huberty 2016 [103] |  |  |  |  | **x** |  |  |  |  | **x** |  |  |  |  |  | Fitbit Flex |
| Jeong 2019 [104] |  |  |  | **x** |  | **x** |  | **x** |  | **x** |  |  |  |  |  | Fitbit Charge |
| Jiang 2019 [105] | **x** |  |  |  |  |  |  |  |  |  |  |  |  |  |  | Fitbit Charge |
| John-Henderson 2019 [106] |  |  |  |  |  |  |  |  |  | **x** | **x** |  |  |  |  | ActiGraph GT9X Link |
| Jones 2020 [107] |  |  |  |  | **x** |  |  |  | **x** |  |  |  |  |  |  | ActiGraph GT3X+ |
| Jonker 2020 [108] |  |  |  | **x** | **x** |  |  |  |  |  |  |  |  |  |  | Fitbit Charge |
| Kabbach 2017 [109] | **x** | **x** |  |  |  |  |  |  |  |  |  |  |  |  |  | Polar chest-strap (+ receiver) |
| Kagamiyama and Yano 2018 [110] |  |  |  | **x** |  | **x** |  | **x** |  | **x** |  |  |  |  |  | Fitbit One |
| Kaura 2019 [111] |  |  | **x** |  |  |  |  |  |  |  |  |  |  |  |  | Zio Patch |
| Kim 2019 [113] |  |  |  |  |  |  | **x** |  |  |  |  |  |  |  |  | Axivity AX3 |
| Kim 2019 [112] |  |  |  | **x** |  | **x** |  | **x** |  |  |  |  |  |  |  | Fitbit Charge |
| Kimura 2020 [114] |  |  |  | **x** |  |  |  |  |  | **x** | **x** |  |  |  |  | Silmee W20 |
| Kochiya 2017 [115] | **x** | **x** |  |  |  |  | **x** |  |  |  |  |  |  |  |  | myBeat |
| Koehler 2018 [116] |  |  | **x** |  |  |  |  |  |  |  |  |  |  |  |  | PhysioMem PM 1000 |
| Kolk 2020 [117] |  |  |  | **x** |  |  |  |  |  |  |  |  |  |  |  | Fitbit Flex |
| Kruizinga 2020 [118] | **x** |  |  | **x** |  |  |  |  |  | **x** |  |  |  |  |  | Withings Steel |
| Lamar 2016 [119] |  |  |  | **x** |  |  |  |  |  |  |  |  |  |  |  | Fitbit One |
| Larsen 2020 [120] |  |  |  |  |  |  |  |  |  | **x** |  |  |  |  |  | Fitbit Charge |
| Lazaridou 2020 [121] |  |  |  | **x** |  |  |  |  |  |  |  |  |  |  |  | Fitbit Flex |
| Lee 2018 [123] | **x** |  |  | **x** |  |  | **x** | **x** |  | **x** | **x** |  |  |  |  | Fitbit Charge |
| Lee 2019 [125] |  |  |  |  | **x** |  |  |  |  |  |  |  |  |  |  | Fitbit One |
| Lee 2020 [124] |  |  |  |  |  |  | **x** |  |  |  |  |  |  |  |  | Xiaomi Mi Band |
| LeHello 2018 [122] |  |  |  | **x** | **x** |  |  |  |  |  |  |  |  |  |  | Withings Pulse |
| Liao 2020 [126] |  |  |  | **x** | **x** |  |  |  |  | **x** |  |  |  |  |  | Fitbit Flex |
| Lim 2018 [127] |  |  |  |  |  |  | **x** |  |  |  |  |  |  |  |  | GENEActiv |
| Lim 2018 [128] | **x** |  |  | **x** |  |  |  |  |  |  |  |  |  |  |  | Fitbit Charge |
| Lin 2020 [129] |  |  |  | **x** |  |  |  |  |  |  |  |  |  |  |  | Apple Watches |
| Liu 2016 [130] |  |  |  |  | **x** |  |  |  |  |  |  |  |  |  |  | ActiGraph AM-7164 |
| Low 2017 [132] |  |  |  | **x** | **x** |  | **x** | **x** | **x** | **x** | **x** |  |  |  |  | Fitbit Charge |
| Low 2018 [131] |  |  |  | **x** |  |  |  |  |  |  |  |  |  |  |  | Fitbit Flex, Charge |
| Ma 2018 [133] |  |  |  | **x** |  |  |  |  |  |  |  |  |  |  |  | Jawbone Up |
| Maijala 2019 [134] |  |  |  |  |  |  |  |  |  |  |  |  | **x** |  |  | Oura ring |
| Makic 2020 [135] | **x** | **x** |  | **x** |  |  |  |  | **x** | **x** | **x** |  |  |  |  | Fitbit Alta |
| Massey 2018 [137] |  |  |  | **x** | **x** |  |  |  |  |  |  |  |  |  |  | Fitbit Flex |
| Massey 2020 [136] |  |  |  |  | **x** |  |  |  |  |  |  |  |  |  |  | Fitbit Flex |
| Matcham 2019 [138] | **x** |  |  |  | **x** |  |  |  | **x** | **x** | **x** |  |  |  |  | Fitbit Charge |
| McLean 2018 [139] |  |  |  | **x** |  |  |  |  |  |  |  |  |  |  |  | Fitbit Flex |
| Mead 2019 [140] |  |  |  | **x** | **x** | **x** |  |  |  | **x** |  |  |  |  |  | Fitbit Flex |
| Meijer 2014 [141] |  |  |  |  | **x** |  | **x** |  |  |  |  |  |  |  |  | CIRO Activity Monitor (now: MOX activity monitor) |
| Melin 2016 [142] |  |  |  |  | **x** |  | **x** |  |  |  |  |  |  |  |  | ActiGraph GT3X |
| Menai 2017 [143] |  |  |  | **x** |  |  |  |  |  |  |  | **x** |  |  |  | Withings Pulse and wireless bp monitor |
| Mendelsohn 2019 [144] | **x** |  |  | **x** |  |  |  | **x** | **x** | **x** |  |  |  |  |  | Fitbit Charge |
| Mičková 2019 [145] |  |  |  | **x** |  | **x** |  | **x** | **x** | **x** | **x** |  |  |  |  | Xiaomi Mi Band |
| Miller 2020 [146] | **x** | **x** |  |  |  |  |  |  |  |  |  |  |  | **x** |  | Whoop strap |
| Mobbs 2016 [147] |  |  |  | **x** |  | **x** |  | **x** |  |  |  |  |  |  |  | Fitbit Zip |
| Mocny-Pachońska 2020 [148] | **x** |  |  |  |  |  |  |  |  |  |  |  |  |  |  | Garmin Vivoactive |
| Modarress-Sadeghi 2019 [149] |  |  |  |  | **x** |  |  |  |  |  |  |  |  |  |  | Polar Active |
| Mora-Gonzalez 2019 [150] |  |  |  | **x** |  |  | **x** |  | **x** |  |  |  |  |  |  | ActiGraph GT3X+ |
| Morcos 2020 [151] |  |  |  | **x** |  |  |  |  |  |  |  |  |  |  |  | Fitbit Flex |
| Morhardt 2017 [152] |  |  |  |  |  |  |  |  |  | **x** |  |  |  |  |  | Fitbit Flex |
| Mueller 2019 [153] | **x** |  |  | **x** | **x** |  |  | **x** | **x** |  |  |  |  |  |  | Apple Watches |
| Murphree 2017 [154] |  |  |  | **x** |  | **x** | **x** |  |  |  |  |  |  |  |  | ActiGraph wGT3X-BT |
| Natarajan 2019 [155] | **x** | **x** |  |  |  |  |  |  |  |  |  |  |  |  |  | Fitbit devices |
| Niemela 2019 [156] |  |  |  |  |  | **x** |  |  |  |  |  |  |  |  |  | Polar Active |
| Nishida 2017 [157] |  |  |  |  |  |  | **x** |  |  |  |  |  |  |  |  | FS-750 Estera Accelerometer |
| Nowell 2019 [158] | **x** |  |  | **x** | **x** | **x** |  | **x** |  | **x** | **x** |  |  |  |  | Fitbit Versa |
| Orme 2019 [159] |  |  |  |  | **x** |  |  |  | **x** | **x** |  |  |  |  |  | ActiGraph wGT3X-BT |
| Otsuki und Ishii 2017 [160] | **x** |  |  |  | **x** | **x** |  |  |  |  |  | **x** |  |  |  | Nissei wrist bp Monitor, Omron Pedometer |
| Pakhomov 2020 [161] | **x** |  |  |  |  |  |  |  |  |  |  |  |  |  |  | Fitbit devices |
| Pastor 2020 [162] | **x** |  |  | **x** |  |  |  | **x** |  | **x** | **x** |  |  |  |  | Fitbit devices |
| Peach 2018 [163] |  |  |  |  |  |  |  |  |  | **x** | **x** |  |  |  |  | Fitbit Flex |
| Pépin 2020 [164] |  |  |  | **x** |  |  |  |  |  |  |  |  |  |  |  | Withings trackers |
| Perez 2019 [15] | **x** |  | **x** |  |  |  |  |  |  |  |  |  |  |  |  | Apple Watches, BioTelemetry's ePatch |
| Phillips 2018 [165] |  |  |  | **x** |  |  |  |  |  |  |  |  |  |  |  | Fitbit One / Zip |
| Pozehl 2018 [166] |  |  |  |  | **x** | **x** |  |  |  |  |  |  |  |  |  | ActiGraph GT3X |
| Pradhan and Kelly 2019 [167] |  |  |  | **x** | **x** |  |  |  |  |  |  |  |  |  |  | Fitbit Charge |
| Quer 2020 [168] | **x** | **x** |  |  |  |  |  |  |  |  |  |  |  |  |  | Fitbit devices |
| Quer 2020 [169] | **x** |  |  | **x** |  |  |  |  |  | **x** |  |  |  |  |  | Fitbit devices |
| Quiroz 2018 [170] | **x** |  |  |  |  |  | **x** |  |  |  |  |  |  |  |  | Polar chest-strap (+ receiver), Samsung Gear watch |
| Radin 2020 [14] | **x** |  |  |  |  |  |  |  |  | **x** |  |  |  |  |  | Fitbit devices |
| Rahman 2020 [172] | **x** |  |  |  |  |  | **x** |  |  |  |  |  |  |  |  | Samsung Gear watch |
| Rahman 2020 [171] | **x** | **x** |  |  |  |  |  |  |  |  |  |  |  |  | **x** | Xiaomi Mi Band |
| Ramirez 2020 [173] | **x** |  |  | **x** |  |  |  |  |  | **x** | **x** |  |  |  |  | Fitbit devices |
| Raywood 2020 [174] | **x** |  |  | **x** |  |  |  |  |  |  |  |  |  |  |  | Fitbit Alta |
| Reddy 2018 [175] |  |  |  |  |  |  |  |  |  | **x** |  |  |  |  |  | ActiGraph wGT3X‐BT |
| Roe 2016 [176] |  |  |  | **x** |  |  |  |  |  |  |  |  |  |  |  | Fitbit Flex |
| Root 2017 [177] |  |  |  | **x** | **x** |  |  |  | **x** |  |  |  |  |  |  | Actigraph GT3X+ |
| Rykov 2020 [178] | **x** |  |  | **x** | **x** |  | **x** | **x** | **x** |  |  |  |  |  |  | Fitbit Charge |
| Saarikko 2020 [179] | **x** |  |  | **x** | **x** |  |  |  |  | **x** |  |  |  |  |  | Garmin Vivosmart |
| Saif 2020 [180] |  | **x** |  |  |  |  |  |  |  | **x** | **x** |  |  |  |  | Whoop strap |
| Scarlett 2020 [181] |  |  |  |  |  |  |  |  |  | **x** |  |  |  |  |  | GENEActiv |
| Sehgal 2019 [182] |  |  |  | **x** | **x** |  |  |  |  |  |  |  |  |  |  | Fitbit Charge |
| Semaan 2020 [183] |  |  |  | **x** |  |  |  |  |  |  |  |  |  |  |  | Fitbit devices |
| Shaw 2019 [184] |  |  |  | **x** |  |  |  | **x** | **x** |  |  |  |  |  |  | Fitbit Alta |
| Shen 2017 [185] |  |  |  |  |  |  | **x** |  |  |  |  |  |  |  |  | ActiGraph GT3 |
| Shilaih 2017 [187] | **x** |  |  |  |  |  |  |  |  |  |  |  |  |  |  | Ava bracelet, PulseOn bracelet |
| Shilaih 2018 [186] |  |  |  |  |  |  |  |  |  |  |  |  | **x** |  |  | Ava bracelet |
| Shufelt 2020 [188] | **x** |  |  | **x** |  |  |  |  |  |  |  |  |  |  |  | Fitbit Charge |
| Silverman-Lloyd 2018 [189] |  |  |  | **x** |  |  |  |  |  |  |  |  |  |  |  | Fitbit Charge |
| Smith 2016 [190] |  |  |  | **x** |  |  |  |  |  | **x** |  |  |  |  |  | Fitbit devices |
| Solomon 2016 [191] |  |  | **x** |  |  |  |  |  |  |  |  |  |  |  |  | Zio Patch |
| Souza 2019 [192] |  |  |  |  | **x** | **x** |  |  | **x** | **x** | **x** |  |  |  |  | GENEActiv Original |
| Steinhubl 2018 [193] |  |  | **x** |  |  |  |  |  |  |  |  |  |  |  |  | Zio Patch |
| Stelzer 2018 [194] |  |  |  | **x** |  |  |  |  |  |  |  |  |  |  |  | Fitbit Zip |
| Strain 2020 [195] |  |  |  |  | **x** | **x** | **x** |  | **x** |  |  |  |  |  |  | Axivity AX3 |
| Tateishi 2014 [196] |  | **x** |  |  |  |  |  |  |  |  |  |  |  |  |  | Polar chest-strap (+ receiver) |
| Thijs 2019 [197] |  |  |  | **x** |  | **x** |  |  |  |  |  |  |  |  |  | Fitbit Charge |
| Thomas 2016 [198] |  |  |  | **x** |  |  |  |  |  |  |  |  |  |  |  | Dynamo Activity Tracker |
| Thompson 2018 [199] | **x** |  |  | **x** |  | **x** |  |  |  |  |  |  |  |  |  | Fitbit Charge |
| Thorup 2016 [200] |  |  |  | **x** |  |  |  |  |  |  |  |  |  |  |  | Fitbit Zip |
| Tomitani 2020 [201] |  |  |  |  |  |  |  |  |  |  |  | **x** |  |  |  | Omron HeartGuide |
| Turakhia 2015 [202] |  |  | **x** |  |  |  |  |  |  |  |  |  |  |  |  | Zio Patch |
| Turel 2016 [203] |  |  |  |  |  |  |  |  |  | **x** |  |  |  |  |  | Fitbit Ultra, One |
| Twiggs 2018 [204] |  |  |  | **x** |  |  |  |  |  |  |  |  |  |  |  | Fitbit Flex |
| Van der Kamp 2019 [205] |  |  |  | **x** | **x** |  |  |  | **x** |  |  |  |  |  |  | Fitbit Zip |
| Van Wamelen 2019 [206] |  |  |  |  |  |  |  |  | **x** |  |  |  |  |  |  | Parkinson’s KinetiGraph |
| Vaughn 2019 [207] |  |  |  | **x** |  |  |  |  |  |  |  |  |  |  |  | Fitbit Zip |
| Venkataramanan 2019 [208] |  |  |  | **x** | **x** | **x** |  | **x** |  | **x** | **x** |  |  |  |  | Fitbit Charge |
| Wallace 2016 [209] |  |  |  | **x** |  |  |  | **x** | **x** |  |  |  |  |  |  | Fitbit Ultra |
| Wang 2020 [210] | **x** |  |  | **x** |  |  |  |  |  | **x** |  |  |  |  |  | Fitbit Charge |
| Weatherall 2018 [211] |  |  |  | **x** |  |  |  |  |  | **x** |  |  |  |  |  | Fitbit Charge |
| Weeks 2018 [212] | **x** |  |  |  |  |  |  |  |  |  |  |  |  |  |  | Fitbit Charge |
| Wiles 2020 [213] | **x** |  |  |  |  |  |  | **x** | **x** |  |  |  |  |  |  | Polar chest-strap (+ receiver) |
| Winnebeck 2018 [214] |  |  |  |  |  |  | **x** |  |  | **x** |  |  |  |  |  | Daqtix Daqtometer |
| Wyatt 2020 [215] | **x** |  | **x** |  |  |  |  |  |  |  |  |  |  |  |  | Apple Watches |
| Xu 2018 [216] |  |  |  | **x** |  |  |  |  |  | **x** |  |  |  |  |  | Fitbit Charge |
| Yang 2019 [217] | **x** |  |  | **x** |  |  |  | **x** | **x** | **x** | **x** |  |  |  |  | Xiaomi Mi Band |
| Youn and Lee 2020 [218] |  |  |  |  | **x** |  | **x** |  |  | **x** | **x** |  |  |  |  | ActiGraph GT9X Link |
| Yu 2020 [219] | **x** |  |  | **x** | **x** |  |  |  | **x** |  |  |  |  |  |  | Fitbit Charge |
| Zhu 2019 [220] |  |  |  |  |  |  | **x** |  |  | **x** |  |  |  |  |  | Axivity AX3 |
| Zhuo 2020 [221] | **x** |  |  |  |  |  |  |  |  |  |  |  |  |  | **x** | Megahealth Curcuil ring |
| No. of studies (n) | **55** | **15** | **12** | **96** | **42** | **23** | **23** | **21** | **26** | **51** | **23** | **4** | **3** | **3** | **2** |  |
