# Supplementary material for: The Impact of Wearable Technologies in Health Research: Scoping Review
Source: JMIR Mhealth Uhealth. 2022 Jan 25;10(1):e34384. doi: 10.2196/34384 (PMC8826148; doi:10.2196/34384)
Supplement: Multimedia Appendix 5 [file mhealth_v10i1e34384_app5.docx]

### Multimedia Appendix 5

**Table 3.** Categorisation of wearable application in the studies

|  | **No. of studies** | **No. of studies (%)** | **No. of participants** | **No. of participants (%)** |
| --- | --- | --- | --- | --- |
| **Correlations – wearable and other physiological data** | 40 | (22%) | 208 235 | (2%) |
| **Population-based research** | 31 | (17%) | 8 381 | (0%) |
| **Outcome assessment** | 30 | (17%) | 1 969 | (0%) |
| **Prognosis, forecasting, risk stratification** | 28 | (16%) | 148 247 | (1%) |
| **Explorative analysis of big datasets** | 10 | (6%) | 9 083 727 | (84%) |
| **Method evaluation** | 40 | (22%) | 1 385 174 | (13%) |
| - Feasibility | 12 | (7%) | 436 | (0%) |
| - Condition, disease monitoring | 8 | (4%) | 2 302 | (0%) |
| - Diagnostics, Screening | 6 | (3%) | 837 935 | (8%) |
| - general, no subgroup applicable | 14 | (8%) | 544 501 | (5%) |

Article references and examples:

#### Correlations – wearable and other physiological data

Kimura et al. [114] correlated steps and sleep measures collected with a wristband in older adults with mild cognitive impairment with measures of Alzheimer disease (neurologic, degenerative disease; measure: cortical amyloid burden and cerebral glucose metabolism). Using “a multiple regression model and change-point regression model”, they found a correlation between sleep time and brain function in their cohort, amongst others. The authors concluded that sleep might be an important lifestyle factor regarding brain function and Alzheimer disease. The authors mentioned that their results might contribute to develop novel interventions including sleep aspects for older patients with mild cognitive impairment.

Shilaih et al. correlated HR [187] and wrist temperature [186] measured by wristbands (of Ava and PulseOn) with menstrual cycle phases (derived by urine ovulation tests measuring a peak in luteinizing hormone). They found significant increases in HR of about two beats-per-minute and an even higher increase of the mid-luteal phase (phase of mid-high concentration of the luteinizing hormone) during the fertile window as compared to the menstrual phase. The results were robust for collected behavioural and nutritional confounders like alcohol consumption and PA. Furthermore, they observed three-day temperature shifts indicating fertility in menstrual cycles, which were robust to collected life style factors. Based on their results, the authors concluded high potential for wearable-based multiparameter fertility awareness methods.

An et al. [48] correlated body fat percentages with several, by wrist-worn Fitbit fitness trackers derived PA measures including steps, sedentary time, PA intensity, and energy expenditure of diabetic patients (Type 2 Diabetes mellitus). For the multiple linear regression analysis, they divided the participants into an exercising and non-exercising group and controlled for age, gender and long-term blood glucose (glycated hemoglobin (HbA1c), as diabetic measure). Amongst others, their correlations suggested that the higher time spent in light intensity PA and/or sedentary, the higher the body fat.

Lazaridou et al. [121] investigated the association of PA and pain in women with fibromyalgia (musculoskeletal chronic pain disorder). They correlated fitness tracker steps (of Fitbit Flex) and outcomes of daily questionnaires assessing pain and symptoms (of fibromyalgia), emotional distress and pain catastrophizing (manner of psychological reaction to pain, part of the fear-avoidance model). Amongst others, a significant correlation between average steps and Fibromyalgia symptoms could be seen, as well as pain catastrophizing as moderator of the association between pain and steps. Authors hypothesised that increases in daily steps (PA) are associated with higher self-reported pain intensity in women with fibromyalgia, particularly in those tending to pain catastrophizing.

In a study population of overweight children, Mora-Gonzalez et al. [150] investigated associations of PA with blood concentrations of three neurotrophic growth factors (brain-derived neurotrophic factor (BDNF), vascular endothelial growth factor (VEGF), and insulin growth factor-1 (IGF-1)). These factors might be important in the cognitive and physical development of children. For PA measures, they used steps, PA intensity (e.g., MVPA) and sedentary time of hip worn accelerometers (ActiGraph GT3X*). Especially one factor (BDNF) correlated with multiple PA measures. Based on their results, particularly light PA like slow walking might stimulate and lead to higher factor levels (BDNF) in overweight children.

Another study [50] tested correlations between PA (measured with a hip-worn Fitbit accelerometer) and scores of questionnaires regarding quality of life, depression and dyspnoea of patients with advanced lung cancer. Higher steps correlated with higher scores of life quality and lower scores in pain and depression.

#### Population-based research:

Hemphill et al. [101] used fitness trackers (Fitbit) for observational and cross-sectional insights in PA of children with congenital heart disease during the COVID-19 pandemic. They observed significant lower steps of children during the pandemic and therefore concluded a potentially higher cardiovascular risk within this population with pre-existing conditions. Also, boys had more steps than girls, and steps decreased, in correspondence to pandemic-related restrictions (of authorities). In both 2019 and 2020, all mean step counts were below guideline-recommended 12 000 steps/day.

Yang et al. [218] looked into PA and sleep (measured by ActiGraph* accelerometers) of Seafarers on ocean duty. Particularly, they measured PA intensity (MVPA) and sleep measures like sleep duration, onset, and waking phase. Also considering daytime, duties and sailing phases, they observed overall low levels of sleep and PA. Off-duty, PA was higher and while sailing, sleep was rather characterized as restless and with more wake periods. Concludingly, the authors recommended PA interventions for seafarers.

Kochiya el al. [115] provided insights into nocturnal HR and HRV of one-year-old infants with a chest patch (myBeat of Union Tool, also measuring accelerometery); their self-developed parameter captured respiratory-related changes of HR and HRV. They observed dynamic changes in the respiratory-related regularity of HR during night. The mean cycle length of the parameter reflecting HRV was shorter than these of adults. They hypothesised that decreased HR and higher (respiratory-related) regularity (parameters) are associated with increased parasympathetic activity.

Eyre et al. [84] observed HR and PA (in combination with a Global Positioning System (GPS) signal) with a chest strap (of Garmin) in children from low-socio-economic environments and different ethnic backgrounds. Next to PA intensity (MVPA), time spent in different environments like in- and outdoors, in green or non-green space, was assessed. Less than half of the children did not meet PA intensity recommendations. Most PA was conducted outdoors. However, children spent little time outdoors, in green spaces and for PA. Therefore, the authors emphasised the importance of outdoor and green spaces to increase PA in children.

Using steps and PA intensity of hip-worn accelerometers (Fitbit), Lee et al. [125] cross-sectionally characterised PA of women in reproductive age in rural Poland. Light-intensity PA prevailed in terms of time spent in PA and steps taken across all age groups. Resultantly, the authors suggested to measure light-intensity PA when assessing habitual PA, which includes domestic labour.

Lamar et al. [119] assessed sedentary behaviour of radiologists (and other physicians), particularly during work with hip-worn accelerometers (Fitbit). Radiologists had the lowest step count of all included physicians and showed more sedentary behaviour. The authors also evaluated different types of rotational work. Compared to other work types, radiologists working in interventional procedures were most active, as per steps and sedentary measurements. Authors emphasised that their results call for workplace interventions.

Wiles et al. [213] characterised HR of adults during recreational skateboarding using an ECG chest strap which also assessed accelerometery (Polar). Participants had a high mean HR (about 70% of predicted age maximum HR) and spent most of the time in MVPA. Authors concluded that skateboarding meets (authorities’) exercise recommendation for cardiovascular fitness and might have implications on community skatepark designs.

Wang et al. [210] generated insights into HR patterns of college students with wrist-worn fitness trackers (Fitbit). Of the 600 students who were observed for two years, the authors saw an increase in mean HR. Social contacts, among other factors, affected HR levels and changes.

#### Outcome Assessment:

Jiang et al. [105] investigated the effect of odour on HR during sleep with wearables (Fitbit). Their analysis also included cortisol and questionnaire assessments, tension and anxiety. HR significantly decreased in association to the odour. The authors concluded that odours via the endocrine and sympathetic nervous system may positively influence stress and psychological constitution, as “the inhalation of citrus ginger aroma during sleep may enhance sympathetic nervous and endocrine system activity while alleviating psychological tension and anxiety”.

The study by Culp and Tonelli et al. [20] used a chest-strap (Zephyr status monitor, Zephyr*) which measured a range of parameters (HR, skin temperature, respiratory rate, kilocalories and PA intensity score). They focused on agricultural workers’ physiological response during agricultural work and related those to three categories of working intensity (mild, moderate and uncomfortable, provided by the chest strap and based on HR) and three classes of climate conditions (mild = < 24 °C, moderate = 24-26.9 °C and uncomfortable ≥ 27 °C, using Wet Bulb Globe Temperature which takes into account wind, barometric pressure, and relative humidity). Authors found significant higher body temperatures, higher HR and respiratory rate within the uncomfortable work intensity category. Authors concluded that strategies for prevention of heat-related illness, as well as awareness training are needed.

In a randomised controlled trial (RCT) by Birkeland et al. [57], investigated the influence of a specific bimanual training intervention on children with unilateral cerebral palsy, a neurologic condition affecting movement and muscle tone. Focusing on effects on the cardiac autonomic regulation system, HR and HRV was measured using an ECG chest strap (Polar), next to other outcomes. The authors concluded effective improvements through the intervention on the cardiac autonomic regulation system and walking endurance of the children.

A clinical trial conducted by Cohen-Holzer et al. [73] investigated the influence of a specific bimanual training intervention on children with unilateral cerebral palsy, a neurologic condition affecting movement and muscle tone. Focusing on effects on the cardiac autonomic regulation system, HR and HRV was measured using an ECG chest strap (Polar), next to other outcomes. Taking also other endpoints into account, the authors concluded effective improvements through the intervention on the cardiac autonomic regulation system and walking endurance of the children.

Tateishi et al. [196] experimentally investigated the effects of interactions between women with neuroticism and their pet dogs. HRV, as quantitative parameter of autonomic regulation, was measured with an ECG chest strap (Polar) during the experimental phases: resting, command-communication with the dog (or non-communication for the control group), separation and free time. Neuroticism questionnaire scores were administered, which indicated a possible vulnerability to daily stress. In the experimental group, participants with HR increases while resting had significantly higher neuroticism scores. Parasympathetic activation, seen through specific changes in HRV, happened in women after command-communication interaction with their dogs. Authors therefore emphasised possible benefits of daily dog interactions for woman with high neuroticism.

#### Prognostic, forecasting:

To enhance prediction of influenza-like illness rates, Radin et al. [14] used de-identified wristband wearable data (Fitbit) of resting HR, sleep time and weekly estimates of influenza-like illness (ILI) rates at the state level (reported by an US authority). Wearable data significantly improved predictions in all states, particularly proportions of users with abnormal data were mostly related with ILIs. Authors emphasised the importance of wearable data for fast outbreak responses.

Low et al. [131] measured PA with a wrist-worn fitness tracker (Fitbit) of inpatient recovering patients after metastatic cancer surgery to predict readmission risk (30- and 60-day) (with multivariate logistic regressions). Steps were expected to be related to symptom burden. Results adjusted for possible confounders like age and comorbidity (age, body mass index, comorbidity, and length of postoperative stay) showed that higher inpatient step counts predicted lower readmission risks.

The study conducted by Miller et al. [146] analysed changes in respiratory rate, amongst others, measured with a wristband wearable (Whoop strap) and self-reported data (via the Whoop commercial application platform) to predict the risk of COVID-19 infection. They were able to identify 20% of cases prior to symptom onset and 80% of the cases by the third day of onset. Authors emphasised the potential for early disease detection, outbreak and self-isolation management.

Also for early COVID-19 detection, the team of Quer et al. [169] successfully used wearable consumer platforms and applications (API of Fitbit, Healthkit, GoogleFit), including HR, steps and sleep time, self-reported symptoms and COVID-19 testing results, and multivariate logistic regression. Wearable data significantly improved detection and prediction as compared to symptoms alone. Such predictive algorithms might complement COVID-19 testing, as per the authors.

Yang et al. [217] used PA, HR and sleep data measured with Fitness trackers (Xiaomi) of community-dwelling elderly for fall-risk classifications, next to a clinical questionnaire. Elderly participants were split in three fall categories with supervised learning approaches, whereby best results were yielded with a combination of wearable and questionnaire data. Findings might help identify elderly at risk of falls to prevent injuries.

#### Explorative analysis of big datasets:

Frie et al. [89] used wrist-worn wearables (Withings) user data, focusing on weight and steps, to explore patterns of weight and PA, as well as to explore individual reasons for stopping weight monitoring. They found that PA and PA monitoring declined and “weight increases, suggesting that declining motivation for weight control and difficulties with making use of negative weight feedback” [89].

With the data of the UK biobank cohort of over 80 000 participants, Zhu et al. [220] explored sleep, PA measured with wrist-worn accelerometers (Axtivity*) and coffee intake, amongst others. They found that short sleepers were older, socially deprived and had high coffee intake and “those who slept 6–7 hours/night were most physically active.” [220].

Pépin et al. [164] used Withings wearable data to track steps of approximately 742 000 users to explore COVID-19 home confinement adherence. They looked into PA of pandemic and pre-pandemic periods, also considering different lockdown measures in different countries. Based on their results, partial lockdown measures without strict home confinement did not significantly impact PA. When compared to Chinese users, Europeans took twice as many steps during lockdown house confinement. An increase of steps was found in the last two weeks of confinement suggesting a decrease of compliance.

Menai et al. [143] explored blood pressure (bp) in correlation to step activity, with the help of Withings user data (secondary data) measured with wrist-worn wearables and wireless bp monitors across 37 countries. Adjusted for age, sex, and a number of wearable measurements, mean daily steps were inversely associated with mean systolic and diastolic bp. They found that an increase in steps from one month to another lead to a decrease of bp in an obese population.

#### Method evaluation:

For example, Vaughn et al. [207] investigated accuracy of patient reported PA with the help of hip-worn accelerometers (Fitbit) as reference. Patients who had joint surgery reported walking lengths, which were compared to accelerometer steps. With a high mean error (of over 50%) and high magnitude errors, patient reports were „neither accurate nor reproducible”.

Another study by Gluck et al. [92] evaluated the use of smartphone steps among survivors of critical illness and compared smartphone recorded steps to two wearables, a wristband (Fitbit fitness tracker) and hip-waist-worn accelerometer (steps, distance), serving as reference. A strong linear relationship could be seen between wearable and smartphone steps which indicated potential for smartphone data to assess PA in critical illness survivors.

The Apple Heart Study [15] evaluated ability and false positive rates of Apple watches irregular pulse algorithm to identify AF. A chest-worn ECG patch (BioTelemetry Inc.*) served as a gold-standard reference to compare outcomes of Apple watches. Irregular pulse notifications were rare (0,5%). AF was recorded in about a third of participants receiving an irregular pulse notification. The authors concluded that the algorithm might be able to correctly identify AF in users with irregular pulse notifications on their Apple watch, although a need for further studies was identified.

##### **Method evaluation - Feasibility**

Saif et al. [180] tested the feasibility of using a wrist-worn wearable (Whoop strap) in patients at risk for Alzheimer's disease, a chronic neurodegenerative disease. They qualitatively explored the feasibility and acceptance of elderly participants towards wearables and aimed to find a pre-dementia phase based on wearable measures as a window of opportunity for interventions. With a clustering algorithm (K-means), they identified two patients risk groups, characterised by age, HRV, sleep and cognitive tests. Participant’s acceptance of the wearable was very high. The authors underlined the feasibility of the wearable for data collection and monitoring of this population.

Another study by Burnett-Zeigler et al. [66] investigated the feasibility and acceptability of a psychological intervention targeting mindfulness where the wristband wearable (Zensorium tracker) measured HR and pulse pressure (difference between systolic and diastolic blood) as part of the intervention. They qualitatively identified challenges participants experienced and found no significant changes in stress after the intervention.

The study by Saarikko et al. [179] explored the feasibility to continuously monitor PA, sleep and HR of pregnant women with a wrist-worn Fitness tracker (Garmin). Valid PA and sleep data was available for most days of pregnancy and postpartum. PA and sleep decreased while HR increased during the pregnancy. Authors concluded high feasibility and a potential for their monitoring system for health care.

##### **Method evaluation – Disease monitoring**

Downey et al. [80] continuously monitored vital signs (HR, respiratory rate and temperature) of patients admitted to surgical wards with a chest patch (SensiumVitals, Sensium*). The transmitted live data were continuously available to medical staff. Monitored patients received antibiotics faster after evidence of sepsis, had shorter hospital stays and less readmissions compared to patients allocated to intermittent monitoring (general practice). Future studies are needed to verify results, as per the authors.

Hamed et al. [99] investigated the use of hip-worn accelerometers to monitor the progression of late-onset Pompe disease, a rare genetic disorder typically causing progressive mobility and breathing difficulties. Factors effecting step counts were age, delay of diagnosis, time lived with disease, need of assistance, and patient-reported pain. According to authors, using wearables might be a valid approach to measure mobility in this population.

Koehler et al. [116] examined efficacy of their telemedical intervention for heart failure patients, including wearable ECG measurements (Physiomem Getemed*). Participants of the telemedical intervention had fewer days lost due to unplanned cardiovascular hospital admissions or all-cause death compared to the control group.

Zhou et al. [221] monitored stress and sleep with a wearable ring measuring oximetry and HR, in combination with questionnaires, among medical and nursing staff in Wuhan, China during the COVID-19 pandemic. Almost 40% of the staff had insomnia based on questionnaire results and showed comorbid sleep apnoea signs in nocturnal oximetry. Authors suggested focusing on comorbidity, as well as recommended insomnia medication in future emergency situations.

##### **Method evaluation – Diagnostics, Screening**

Steinhubl et al. [193] evaluated the screening for AF patients with an ECG patch (Zio patch by IRhythm*). Using the wearable screening led to significantly higher AF detections compared to the control group, higher initiation of anticoagulants and higher outpatient physician visits. Therefore, authors highlighted the potential of wearable screening for AF.

Using the same ECG patch (Zio patch*), Bolourchi et al. [61] evaluated the diagnosis of arrhythmia in children with different indications for ECG monitoring for a duration of two weeks. The screening showed relatively high rates of arrhythmias in children. From patch application to first detection of arrhythmias (as well as first symptom-triggered arrhythmias) about 3 days passed in average. Authors therefore concluded a possible advantage of using wearables for arrhythmia screening as compared to the 48h standard monitoring.

Gou et al. [97] evaluated screening for AF in high-risk patients (identified by a score assessing cardio-vascular risk factors) compared to population-wide screening. Authors used data of different wrist-worn Huawei wearables with in-built AF detection based on pulse measurement and data of symptom questionnaires. Highest detection rates were the result of a combination of symptoms (palpitations) and the cardio-vascular risk score. Findings might increase use of wearables for AF screening and prevention.

Wyatt et al. [215] retrospectively evaluated the in-built pulse evaluation of Apple watches for abnormal pulse detection. Clinical patients were included if they had presented with watch notifications and received a respective clinical evaluation. Only few patients who received a notification were diagnosed with a clinically relevant cardiovascular diagnosis. Therefore, authors warned of high false positive notifications which may result in health care overutilization.
